# KIR3DL01 upregulation on gut natural killer cells in response to SIV infection of KIR- and MHC class I-defined rhesus macaques

**DOI:** 10.1371/journal.ppat.1006506

**Published:** 2017-07-14

**Authors:** Moritz Ries, Matthew R. Reynolds, Ksenia Bashkueva, Kristin Crosno, Saverio Capuano, Trent M. Prall, Roger Wiseman, David H. O’Connor, Eva G. Rakasz, Hajime Uno, Jeffrey D. Lifson, David T. Evans

**Affiliations:** 1 Department of Pathology and Laboratory Medicine, University of Wisconsin-Madison, Madison, Wisconsin, United States of America; 2 Wisconsin National Primate Research Center, University of Wisconsin-Madison, Madison, Wisconsin, United States of America; 3 Department of Biostatistics and Computational Biology, Dana-Farber Cancer Institute, Boston, Massachusetts, United States of America; 4 AIDS and Cancer Virus Program, Leidos Biomedical Research Inc., Frederick National Laboratory for Cancer Research, Frederick, Maryland, United States of America; Emory University, UNITED STATES

## Abstract

Natural killer cells provide an important early defense against viral pathogens and are regulated in part by interactions between highly polymorphic killer-cell immunoglobulin-like receptors (KIRs) on NK cells and their MHC class I ligands on target cells. We previously identified MHC class I ligands for two rhesus macaque KIRs: KIR3DL01 recognizes Mamu-Bw4 molecules and KIR3DL05 recognizes Mamu-A1*002. To determine how these interactions influence NK cell responses, we infected KIR3DL01^+^ and KIR3DL05^+^ macaques with and without defined ligands for these receptors with SIV_mac_239, and monitored NK cell responses in peripheral blood and lymphoid tissues. NK cell responses in blood were broadly stimulated, as indicated by rapid increases in the CD16^+^ population during acute infection and sustained increases in the CD16^+^ and CD16^-^CD56^-^ populations during chronic infection. Markers of proliferation (Ki-67), activation (CD69 & HLA-DR) and antiviral activity (CD107a & TNFα) were also widely expressed, but began to diverge during chronic infection, as reflected by sustained CD107a and TNFα upregulation by KIR3DL01^+^, but not by KIR3DL05^+^ NK cells. Significant increases in the frequency of KIR3DL01^+^ (but not KIR3DL05^+^) NK cells were also observed in tissues, particularly in the gut-associated lymphoid tissues, where this receptor was preferentially upregulated on CD56^+^ and CD16^-^CD56^-^ subsets. These results reveal broad NK cell activation and dynamic changes in the phenotypic properties of NK cells in response to SIV infection, including the enrichment of KIR3DL01^+^ NK cells in tissues that support high levels of virus replication.

## Author summary

Natural killer (NK) cells are an important cellular defense against viral pathogens, and are regulated in part by interactions between killer-cell immunoglobulin-like receptors (KIRs) on NK cells and MHC class I ligands on target cells. Using multi-parameter flow cytometry, we report the first longitudinal study of changes in the phenotypic and functional properties of NK cells in KIR- and MHC class I-defined rhesus macaques infected with simian immunodeficiency virus (SIV). Our findings reveal broad NK cell activation and highly dynamic changes in the phenotypic properties of NK cells in response to SIV infection, including an enrichment of NK cells expressing KIR3DL01 in tissues that represent sites of high levels of virus replication.

## Introduction

Natural killer cells provide a critical early defense against viral pathogens by directly responding to infected cells without prior antigenic stimulation. This is accomplished through the integration of signals from activating and inhibitory receptors, which in primates include the highly polymorphic killer-cell immunoglobulin-like receptors (KIRs) [1,2]. KIRs contain two or three extracellular immunoglobulin-like domains (2D or 3D), and depending on whether they have long (L) or short (S) cytoplasmic tails, transduce either inhibitory or activating signals [1,2]. MHC class I molecules serve as ligands for the inhibitory KIRs [1,2], and although the ligands for the activating KIRs are not as well defined, there is evidence that these receptors also recognize MHC class I molecules [3–5]. In the case of inhibitory KIRs, engagement of ligands on the surface of healthy cells normally suppresses NK cell activation; however, if these interactions are disrupted, for instance as a consequence of MHC class I downregulation by the HIV-1 Nef protein [6–8], this inhibition is lost, triggering NK cell degranulation and the cytolysis of infected cells.

The specificity of inhibitory KIRs is primarily determined by contacts with the α1 and α2 domains of their ligands. All HLA-B molecules and some HLA-A molecules can be categorized as either Bw4 or Bw6 allotypes depending on residues 77–83 of their α1 domains [9]. Whereas KIR3DL1 selectively binds to HLA-Bw4 ligands, no human KIRs are known to recognize HLA-Bw6 molecules. HLA-C molecules can likewise be classified as C1 or C2 allotypes on the basis of polymorphisms at positions 77 and 80, which are recognized respectively by KIR2DL2 and KIR2DL3 or KIR2DL1 depending on the amino acid residues at these positions [10,11]. Consistent with crystal structures showing that KIRs contact HLA class I surfaces over C-terminal peptide residues [12–14], peptides bound by MHC class I ligands can also influence these interactions [15,16].

*KIR* and *HLA class I* polymorphisms are associated with differences in the course of HIV-1 infection [17–19]. In HIV-1 infected individuals, *KIR3DS1* and highly expressed *KIR3DL1* alleles in combination with *HLA-Bw4* alleles encoding isoleucine at position 80 (HLA-Bw4-80I) are associated with lower viral loads and slower courses of disease progression [17,20]. Accordingly, KIR3DS1^+^ and KIR3DL1^+^ NK cells preferentially expand in response to HIV-1 infection in HLA-Bw4-80I^+^ individuals [21]. *In vitro* studies have also shown that KIR3DS1^+^ NK cells can suppress HIV-1 replication in lymphocytes from HLA-Bw4-80I^+^ donors, but not from *HLA-Bw6* homozygous donors [3], and that KIR3DL1^+^ NK cells respond to HIV-1-infected cells that have downmodulated HLA-Bw4 ligands in a manner that reflects hierarchical differences in their education [22]. Additional studies have identified HIV-1 polymorphisms that suppress KIR2DL2^+^ and KIR2DL3^+^ NK cell responses to virus-infected or peptide-pulsed cells *in vitro*, suggesting that HIV-1 is under selective pressure in certain individuals to acquire changes in epitopes that stabilize HLA-C interactions with inhibitory KIRs as a mechanism of immune evasion [23–25].

Simian immunodeficiency virus (SIV) infection of the rhesus macaque is an important animal model for HIV-1 pathogenesis and AIDS vaccine development [26]; however, studies to address the role of NK cells in this system have been limited by immunogenetic differences between humans and macaques and a lack of defined ligands for macaque KIRs. Unlike humans, which have *HLA-A*, -*B* and–*C* genes, macaques and other Old world monkeys do not have a *C* locus [27,28]. Instead, these species have an expanded repertoire of *A* and *B* genes [27–30]. There are up to four *Ma**caca* *mu**latta (Mamu)-A* genes and a highly variable number of *Mamu-B* genes on any given haplotype in the rhesus macaque [31,32]. Macaques accordingly lack *KIR2DL/S* genes that encode receptors for HLA-C, but have an expanded complement of highly polymorphic *KIR3DL/S* genes [33–37]. Phylogenetic and segregation analyses support the existence of 22 *KIR* genes in macaques [35,36,38]; however, as a consequence of the rapid pace of *KIR* evolution, only two of these genes (*Mamu-KIR2DL04* and -*KIR3DL20*) have recognizable human orthologs [1,2,39–41]. Thus, it is not possible to predict the ligands for macaque KIRs based on sequence similarity with their human counterparts.

MHC class I ligands have nevertheless been identified experimentally for a few rhesus macaque KIRs [29,30,42]. Mamu-A1*002, a molecule with a canonical Bw6 motif, was identified as a ligand for Mamu-KIR3DL05 (KIR3DL05) [30]. Mamu-A1*002 and KIR3DL05 are respectively expressed by approximately 20% and 40% of Indian-origin rhesus macaques, and the binding of KIR3DL05 to Mamu-A1*002 is strongly influenced by SIV peptides [16,30]. Functional assays with primary NK cells also identified multiple Bw4 molecules as ligands for Mamu-KIR3DL01 (KIR3DL01). KIR3DL01 is the most polymorphic KIR in rhesus macaques and is expressed by 85–95% of animals of Indian origin [29,40]. Most rhesus macaques also have one or more *Mamu-Bw4* alleles predicted to encode ligands for this receptor. Despite their coincidental similarity in nomenclature, rhesus KIR3DL01 and human KIR3DL1 are not orthologous gene products; however, their shared specificity for Bw4 ligands suggests that they may serve similar functions.

In the present study, we investigated NK cell responses to SIV infection of *KIR-* and *MHC class I*-defined macaques. Twelve *KIR3DL05*^+^ macaques, of which half were Mamu-A1*002^+^, eleven were *KIR3DL01*^+^, and all but one were *Mamu-Bw4*^+^, were infected with SIV_mac_239, and longitudinal changes in NK cell subsets were monitored in peripheral blood and tissues. Infection with SIV broadly stimulated NK cell responses, resulting in significant increases in the number of NK cells in blood expressing markers of activation, proliferation and antiviral activity. Significant increases were also observed in the frequency of KIR3DL01^+^ NK cells in lymph nodes and gut-associated lymphoid tissues. These results reveal dynamic changes in the phenotypic and functional properties of NK cells in response to SIV infection and an enrichment of KIR3DL01^+^ NK cells at sites of early virus replication and CD4^+^ T cell turnover.

## Results

### SIV infection of KIR- and MHC class I-defined rhesus macaques

We previously identified MHC class I ligands for two rhesus macaque KIRs. We found that KIR3DL05 binds to Mamu-A1*002, a common MHC class I molecule in the rhesus macaque with a Bw6 motif [30], and that KIR3DL01, which is among the most polymorphic and commonly expressed KIRs in rhesus macaques, recognizes MHC class I ligands with a Bw4 motif [29]. We further demonstrated that nearly a third of the SIV peptides bound by Mamu-A1*002 suppress the cytolytic activity of KIR3DL05^+^ NK cells by stabilizing this interaction [16]. To determine how these receptor-ligand interactions influence NK cell responses and the outcome of immunodeficiency virus infection, twelve *KIR-* and *MHC class I*-defined rhesus macaques were infected intravenously with SIV_mac_239, and longitudinal changes in NK cells and viral loads were monitored in peripheral blood and lymphoid tissues.

KIR3DL05^+^ macaques were initially identified by staining PBMCs with Mamu-A1*002 Gag GY9 tetramers as previously described [30]. Six Mamu-A1*002^+^ and six–A1*002^-^ animals were then selected from this group on the basis of *MHC class I* genotyping (Table 1). Five of the Mamu-A1*002^+^ animals and three of Mamu-A1*002^-^ animals were also positive for Mamu-A3*13, which encodes another molecule identified as a ligand for KIR3DL05 (Table 1) [42]. As a reflection of the high prevalence of *KIR3DL01* in rhesus macaques, eleven of these animals expressed KIR3DL01 allotypes that could be detected by staining with the NKVFS1 antibody (Table 2) [29]. Eleven of the animals were also positive for one or more *Mamu-Bw4* alleles predicted to encode ligands for KIR3DL01 (Table 1). Complete *KIR* genotyping by next generation sequencing corroborated the presence or absence of *KIR3DL01* and *KIR3DL05*, and identified twenty-three novel *KIR* alleles in these animals (Table 2 & S1 Table).

**Table 1:** MHC class I genotypes.

	rh2547	rh2548	rh2549	rh2550	r09015	rh2552	rh2553	rh2554	rh2555	rh2556	rh2557	rh2558
												
***Mamu-A***	A1*006	A1*002	A1*006	A1*002	A1*002	A1*002	A1*004	A1*006	A1*002	A1*019	A1*004	A1*002
	A1*026	A1*008	A1*023	A1*006	A1*008	A1*008	A1*019	A1*008	A1*008	A1*008	A1*008	A1*007
	A2*05	A2*05	A2*05	A2*05	A2*05	A2*05	A2*05	A2*05	A2*05	A2*05	A2*05	A2*05
	A4*14	A3*13	A4*14	A3*13	A3*13	A3*13	A4*14	A3*13	A3*13	A3*13	A3*13	A4*14
				A4*14		A4*14		A4*14	A4*14	A4*14	A4*14	A6*01
***Mamu-B***	B*019	B*005	B*019	B*001	B*038	B*005	B*001	B*015	B*005	B*001	B*036	B*019
	B*024	B*015	B*024	B*007	B*046	B*015	B*007	B*019	B*015	B*005	B*037	B*024
	B*046	B*019	B*046	B*019	B*047	B*044	B*030	B*024	B*019	B*007	B*045	B*030
	B*051	B*024	B*051	B*024	B*052	B*060	B*052	B*046	B*024	B*015	B*046	B*031
	B*054	B*044	B*057	B*030	B*054	B*070	B*054	B*051	B*044	B*030	B*050	B*043
	B*057	B*046	B*072	B*046	B*055	B*072	B*055	B*057	B*046	B*044	B*051	B*046
	B*060	B*051	B*082	B*051	B*058	B*109	B*057	B*060	B*051	B*057	B*052	B*051
	B*070	B*057	B*109	B*057	B*063		B*058	B*068	B*057	B*060	B*054	B*057
	B*072	B*060		B*060	B*072		B*063	B*072	B*060	B*063	B*055	B*072
	B*074	B*070		B*072	B*082		B*097	B*082	B*070	B*070	B*058	B*073
	B*082	B*072		B*082	B*097		B*098	B*109	B*072	B*072	B*060	B*082
	B*089	B*082		B*109	B*098		B*188		B*082	B*109	B*063	B*092
	B*093	B*109		B*188					B*109	B*188	B*072	B*109
	B*098										B*097	
	B*109										B*098	

Alleles of the *Mamu-A* and–*B* genes (left column) identified in each of the rhesus macaques included in this study are listed below the corresponding animal identification numbers (top row). Alleles predicted to encode ligands for KIR3DL01 (*Mamu-Bw4*) and KIR3DL05 (Mamu-A1*002 and–A3*13) are indicated in bold.

**Table 2:** KIR genotypes.

	rh2547	rh2548	rh2549	rh2550	r09015	rh2552	rh2553	rh2554	rh2555	rh2556	rh2557	rh2558
***KIR1D***		*002	*002	*002	*002	*003:*01*	*002					*003:*02*
***KIR2DL04***	*015:01 *023	*001:*01*	*021	*001:*01*	*001:*01*	*001:01 *023	*012 *023	*021	*015:01 *015:*02*	*015:*01*	*008:02 *022	*015:*01*
***KIR3DL01***	*019:*01*	*002 *003 *018		*002 *012	*002 *012	*016	*019:*01*	*019:*01*	*015	*015	*002 *0016	*015
***KIR3DL02***	*004:*01*							*003	*004:*01*	*004:*01*		*004:*01*
***KIR3DL05***	*007	*007	*007	*008	*015	*007	*007	*008	*006:*01*	*002 *008	*016	*007 *008
***KIR3DL07***			*004	*003	*003			*004	*024	*003 *009:01 *024	*009:*01*	*003 *024
***KIR3DL08***		*002 *007	*007			*007					*007	
***KIR3DL11***	*001		*001			*001	*001					
***KIR3DL20/KIR2DL05***		*027				*026		*012 *014			*011 *025	
***KIR3DS01***	*001:*02*							*005	*001:*02*	*001:*02*		
***KIR3DS02***		*015:*01*	*015:*02*	*009								
***KIR3DS03***		*001:01 *004	*004									
***KIR3DS04***	*007 *008					*007 *008	*007 *008	*008				*001:*02*
***KIR3DS05***		*004		*001 *002:*01*	*002:*01*	*002:*01*					*002:01 *006	
***KIR3DS06***	*011			*002:*02*		*011	*011	*010				
***KIR3DSw07***	*003					*003	*003					
***KIR3DSw09***	*003							*003				

The alleles of rhesus macaque *KIR* genes (left column) identified in each of the macaques included in this study are listed below the corresponding animal identification numbers (top row). Newly identified *KIR* alleles are indicated in bold and their GenBank accession numbers are provided in S1 Table. Gene and allele identifiers correspond to official Immuno-Polymorphism Database designations for rhesus macaque *KIR* sequences.

**Fig 1:**
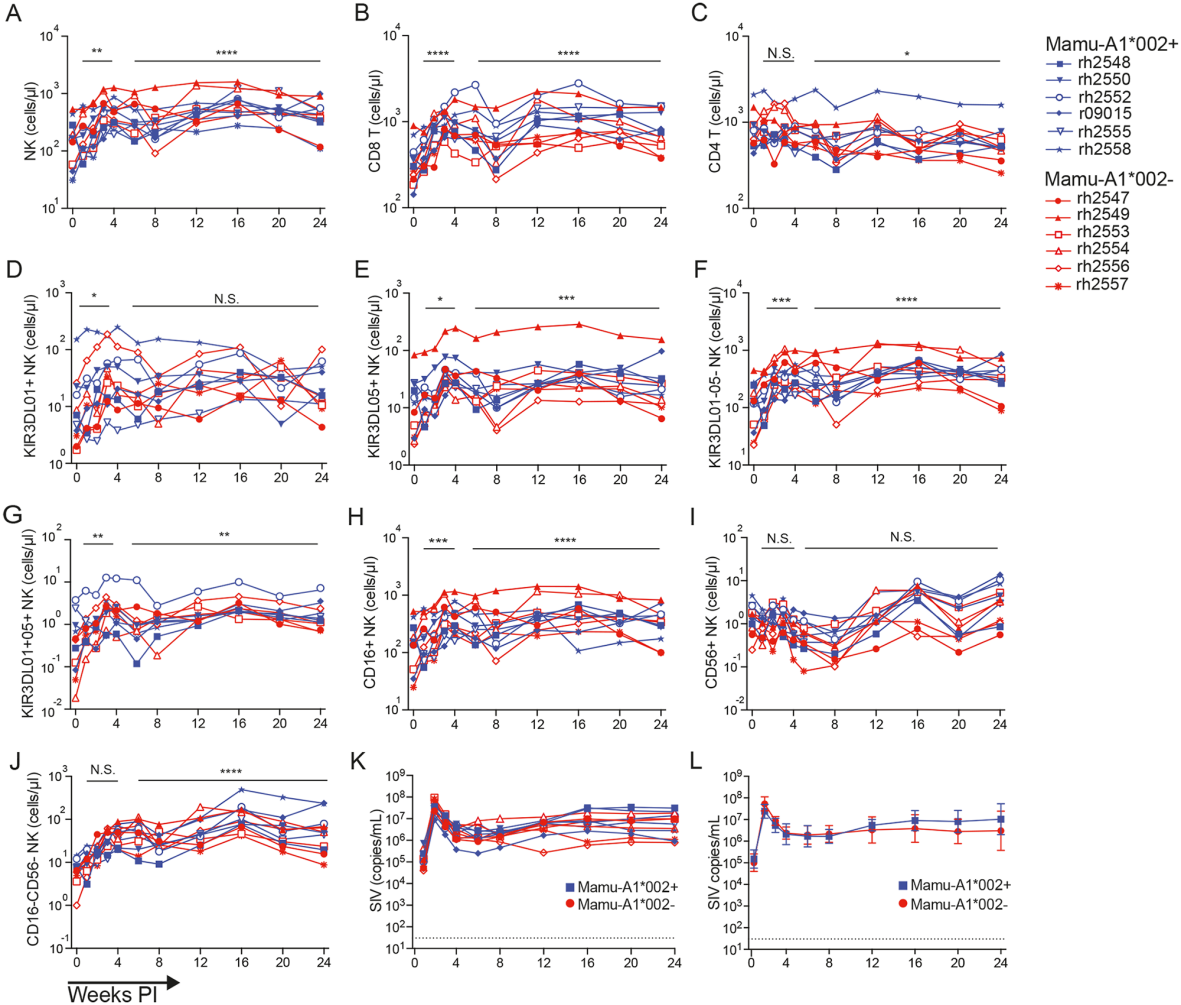
Longitudinal changes in NK and T cell counts in peripheral blood in response to SIV infection. Absolute counts for total NK cells (A), CD8^+^ T cells (B), CD4^+^ T cells (C), KIR3DL01^+^ NK cells (D), KIR3DL05^+^ NK cells (E), KIR3DL01^-^05^-^ NK cells (F), KIR3DL01^+^05^+^ NK cells (G), CD16^+^ NK cells (H), CD56^+^ NK cells (I), and CD16^-^CD56^-^ NK cells (J) were monitored by staining whole blood and PBMCs as described in the methods. Individual (K) and geometric mean (L) SIV RNA loads in plasma are shown for Mamu-A1*002^+^ (blue) and–A1*002^-^ (red) animals. Gating strategies for determining absolute lymphocyte counts in blood and the percentages of PBMCs expressing CD16, CD56, KIR3DL01 and KIR3DL05 are shown in S1 and S4 Figs. Viral loads were measured using a qRT-PCR assay with a detection threshold of 30 copies/ml (dotted line) and error bars indicate 95% CI for geometric mean values. Statistics were calculated using a mixed effects model by comparing results from acute (week 1–4) and chronic (weeks 6–24) infection to pre-infection (week 0) (p<0.05 *, p<0.01**, p< 0.005*** & p<0.001****).

SIV loads in plasma and lymphocyte counts in peripheral blood were monitored at weekly to monthly intervals after SIV inoculation. Absolute counts for NK and T cell subsets were determined by staining whole blood with antibodies to lineage-specific markers in a bead-based assay to adjust for sample volume (S1 Fig). NK cells were defined as CD8^+^CD3^-^ lymphocytes and verified by staining with an antibody to human NKG2A that cross-reacts with multiple NKG2 family members in macaques [43]. PBMCs were also stained in parallel with a separate panel that included antibodies (or tetramer) to KIR3DL01, KIR3DL05 (Mamu-A1*002 Gag GY9 tetramer), CD16 and CD56. Absolute counts for NK cell subsets defined by the expression of KIR3DL01, KIR3DL05, CD16 and CD56 were calculated as a percentage of total NK cell counts at each time point. Longitudinal changes in lymphocyte counts for individual animals are shown in Fig 1 and summarized as mean cell counts for Mamu-A1*002^+^ versus–A1*002^-^ animals in S2 Fig.

Consistent with the variegated expression of KIRs, the majority of NK cells were KIR3DL01/05 double-negative (KIR3DL01^-^05^-^) and there was considerable animal-to-animal variation in the frequency of KIR3DL01^+^ and KIR3DL05^+^ NK cells. Prior to SIV inoculation, KIR3DL01^+^ cells constituted 12.8% ±4.7 (22.5±13.2 cells/μl), KIR3DL05^+^ cells constituted 8.7% ±1.4 (17.3±6.3 cells/μl) and double-positive (KIR3DL01^+^05^+^) cells constituted 0.58% ±0.21 (0.84±0.34 cells/μl) of circulating NK cells. In response to SIV infection, sharp increases were observed in total NK and CD8^+^ T cell counts (Fig 1A and 1B). These responses were reflected by significant increases in each of the KIR-defined NK cell subsets during acute infection (weeks 1–4), and were sustained by the KIR3DL05^+^, KIR3DL01^+^05^+^ and KIR3DL01^-^05^-^ subsets during chronic infection (weeks 6–24) (Fig 1D–1G).

Phenotypic analyses previously defined CD16^+^CD56^-^ and CD16^-^CD56^+^ NK cell populations in rhesus macaques that correspond to CD16^+^CD56^dim^ and CD16^-^CD56^bright^ populations in humans [44,45]. Similar to their human counterparts, CD16^+^CD56^-^ (CD16^+^) NK cells represent the predominant NK cell population in blood and have a higher capacity for cytolytic activity than the less frequent and less mature CD16^-^CD56^+^ (CD56^+^) subset [44]. Macaques also have a CD16^-^CD56^-^ NK cell population that is not found in humans, which appears to represent an intermediate in the differentiation of CD56^+^ NK cells into CD16^+^ NK cells [44–46]. Consistent with a previous cross-sectional study [44], we observed significant increases in cell counts for the CD16^+^ subset during acute and chronic infection (Fig 1H), and for the CD16^-^CD56^-^ subset during chronic infection (Fig 1J), whereas the CD56^+^ population remained unchanged (Fig 1I).

**Fig 2:**
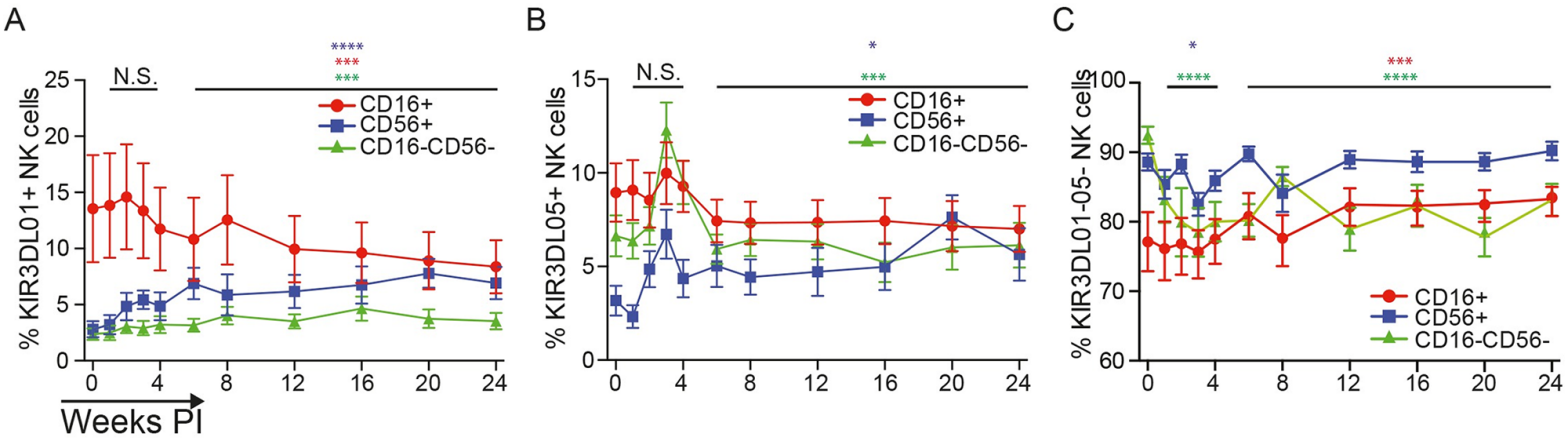
Changes in the frequency of CD16^+^, CD56^+^ and CD16^-^CD56^-^ NK cells expressing KIR3DL01 and KIR3DL05. The percentages of CD16^+^, CD56^+^ and CD16^-^CD56^-^ NK cells that are KIR3DL01^+^ (A), KIR3DL05^+^ (B) or KIR3DL01^-^05^-^ (C) were monitored in peripheral blood. The mean and standard error (error bars) are shown for each subset. Significance values were calculated using a mixed effects model by comparing the results from acute (weeks 1–4) and chronic (weeks 6–24) infection to pre-infection (week 0) and are indicated with asterisks color-coded according to the corresponding NK cell population (p<0.05*, p<0.01**, p< 0.005*** & p<0.001****).

Unlike humans, which only express KIRs on functionally mature CD16^+^CD56^dim^ NK cells, macaques express KIRs on CD16^+^, CD56^+^ and CD16^-^CD56^-^ NK cells [47]. Longitudinal comparisons revealed differences in the distribution of KIR3DL01 and KIR3DL05 on these subsets. Prior to SIV inoculation, KIR3DL01 and KIR3DL05 were both expressed at a higher frequency on CD16^+^ NK cells than on CD56^+^ or CD16^-^CD56^-^ NK cells (Fig 2). Following SIV infection, the frequency of CD56^+^ and CD16^-^CD56^-^ NK cells expressing KIR3DL05 rapidly increased, approaching similar percentages as the CD16^+^ subset during chronic infection (Fig 2B). In contrast, the distribution of KIR3DL01 did not change during acute infection; however, the percentage of KIR3DL01^+^ CD16^+^ NK cells gradually declined coincident with an increase in the frequency of CD56^+^ and CD16^-^CD56^-^ NK cells expressing this KIR during chronic infection (Fig 2A). These changes are reflected by a decrease in the frequency of CD16^-^CD56^-^ NK cells and an increase in the frequency of CD16^+^ NK cells lacking both KIR3DL01 and KIR3DL05 (KIR3DL01^-^05^-^) (Fig 2C). These observations reveal differential changes in the proportion of CD16^+^, CD56^+^ and CD16^-^CD56^-^ NK cells expressing KIR3DL01 versus KIR3DL05 in response to SIV infection.

**Fig 3:**
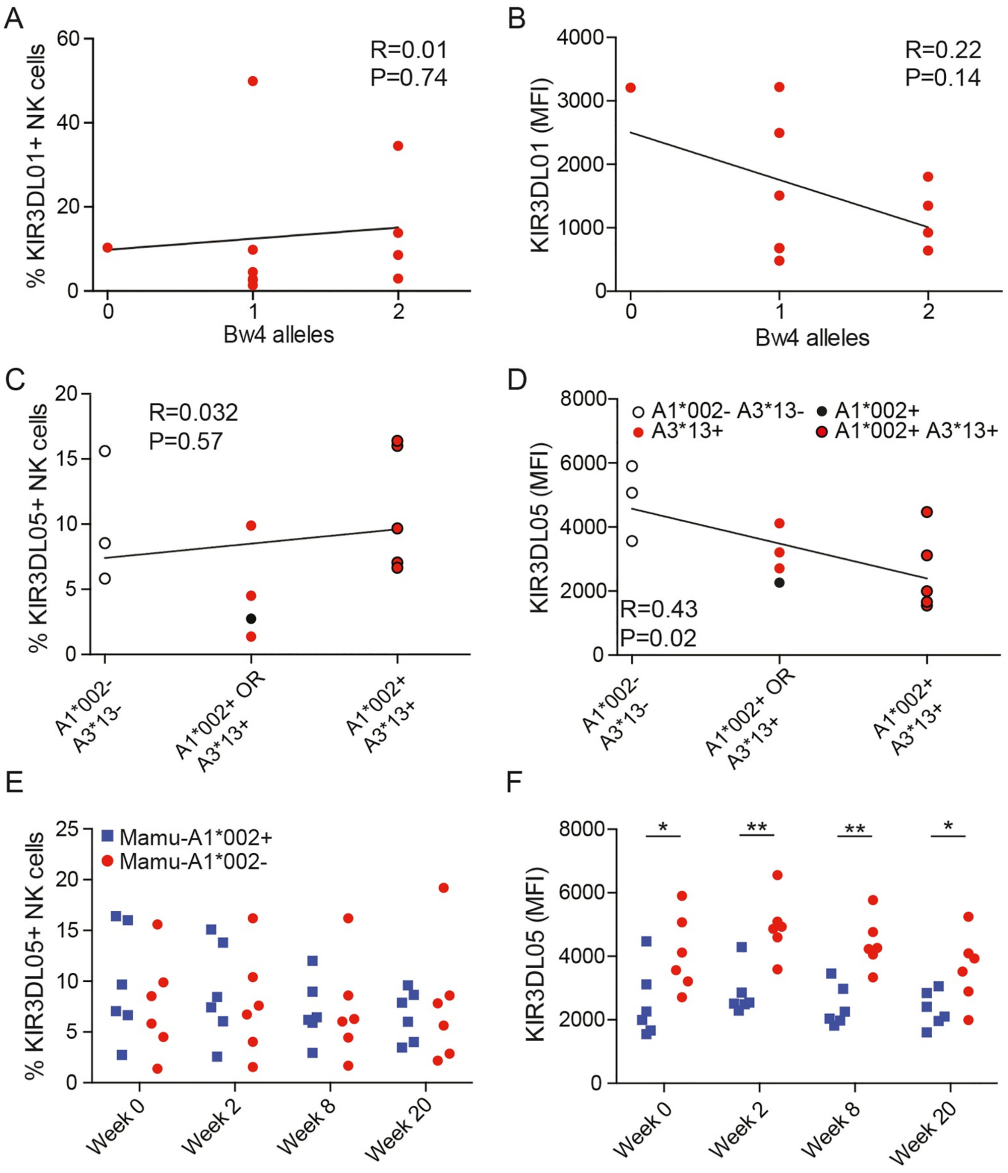
Frequency and intensity of KIR expression as a function of the presence of MHC class I ligands. Comparison of the frequency (A) and the mean fluorescence intensity (B) of KIR3DL01 staining on NK cells prior to SIV infection versus the number of MHC class I alleles predicted to encode Mamu-Bw4 ligands for this receptor by linear regression analysis. Comparison of the frequency (C) and the mean fluorescence intensity (D) of KIR3DL05 staining on NK cells for Mamu-A1*002^-^, -A3*13^-^ (open circle), Mamu-A1*002^-^, -A3*13^+^ (red dot), Mamu-A1*002^+^, -A3*13^-^ (black dot), and Mamu-A1*002^+^, -A3*13^+^ (black circle, red dot) animals prior to SIV infection by linear regression analysis. Comparison of the frequency (E) and the mean fluorescence intensity (F) of KIR3DL05 staining on NK cells for Mamu-A1*002^+^ (blue) versus -A1*002^-^ (red) animals at the indicated time points before and after SIV infection. Significant differences are indicated with asterisks (p<0.05* & p<0.01**, Mann-Whitney *U*-test).

NK cell education in humans increases the frequency of cells bearing KIRs that recognize HLA class I ligands, while decreasing cognate receptor levels on the cell surface [48–50]. We therefore analyzed the percentage NK cells expressing KIR3DL01 and KIR3DL05, and the mean fluorescence intensity of surface staining for these receptors, with respect to the presence or absence of their MHC class I ligands. Neither the frequency nor the intensity of KIR3DL01 staining correlated with the predicted number of *Mamu-Bw4* alleles (Fig 3A and 3B). The frequency of KIR3DL05^+^ NK cells also did not correlate with the number of alleles encoding ligands for this receptor (Mamu-A1*002 and/or–A3*13) (Fig 3C); however, KIR3DL05 staining did correlate inversely with the presence of these alleles (Fig 3D). This correlation appears to be primarily driven by Mamu-A1*002, since independent comparisons of KIR3DL05 staining for animals with or without these alleles revealed significantly lower KIR3DL05 levels in association with Mamu-A1*002 (Fig 3F), but not Mamu-A3*13 (S3A Fig). Additional analyses indicated that these patterns of KIR3DL01 and KIR3DL05 staining did not change in response to SIV infection. Comparisons of the frequency and intensity of KIR3DL01 staining at several time points before and after SIV infection did not reveal significant associations with the number of *Mamu-Bw4* alleles (S3B Fig). Similarly, whereas there was no difference in the frequency of KIR3DL05^+^ NK cells before or after SIV infection (Fig 3E), KIR3DL05 staining was significantly lower for Mamu-A1*002^+^ animals prior to SIV inoculation and at multiple time points during acute and chronic infection (Fig 3F).

The absence of a correlation between KIR3DL01 staining and the number of *Bw4* alleles in these animals may reflect incomplete knowledge of the ligands for this receptor, since several of the *Mamu-Bw4* alleles listed in Table 1 were predicted to encode ligands for KIR3DL01 based on sequences in their α1 and α2 domains [29], rather than on experimental verification. Furthermore, because next generation sequencing methods used for KIR and MHC class I genotyping cannot differentiate two or more alleles with the same sequence, these analyses do not account for possible differences in the copy number of *KIR3DL01* or *Mamu-Bw4* genes that may influence KIR3DL01 expression in some animals. In the case of KIR3DL05, a dominant effect of Mamu-A1*002 on the expression of this receptor is consistent with the unusually high avidity of Mamu-A1*002 for KIR3DL05 [30] and with higher expression levels for Mamu-A1*002 than for ‘minor’ alleles of the Mamu-A3*13 locus [51]. Thus, the lower levels of KIR3DL05 staining detected on NK cells from Mamu-A1*002 animals suggest that this molecule may have a particularly strong effect on the education of KIR3DL05^+^ NK cells.

Despite differences in KIR3DL05^+^ staining in Mamu-A1*002^+^ versus–A1*002^-^ macaques, and changes in the frequency of NK cells expressing this KIR during acute infection, viral loads and CD4^+^ T cell counts did not differ for Mamu-A1*002^+^ versus -A1*002^-^ animals (Fig 1C, 1K and 1L). These comparisons therefore did not reveal an advantage to SIV replication in Mamu-A1*002^+^ macaques as a consequence of the presentation of inhibitory peptides to KIR3DL05^+^ NK cells, as suggested by cell culture experiments with sorted KIR3DL05^+^ NK cells [16].

### Phenotypic profile of KIR-defined NK cell subsets

**Fig 4:**
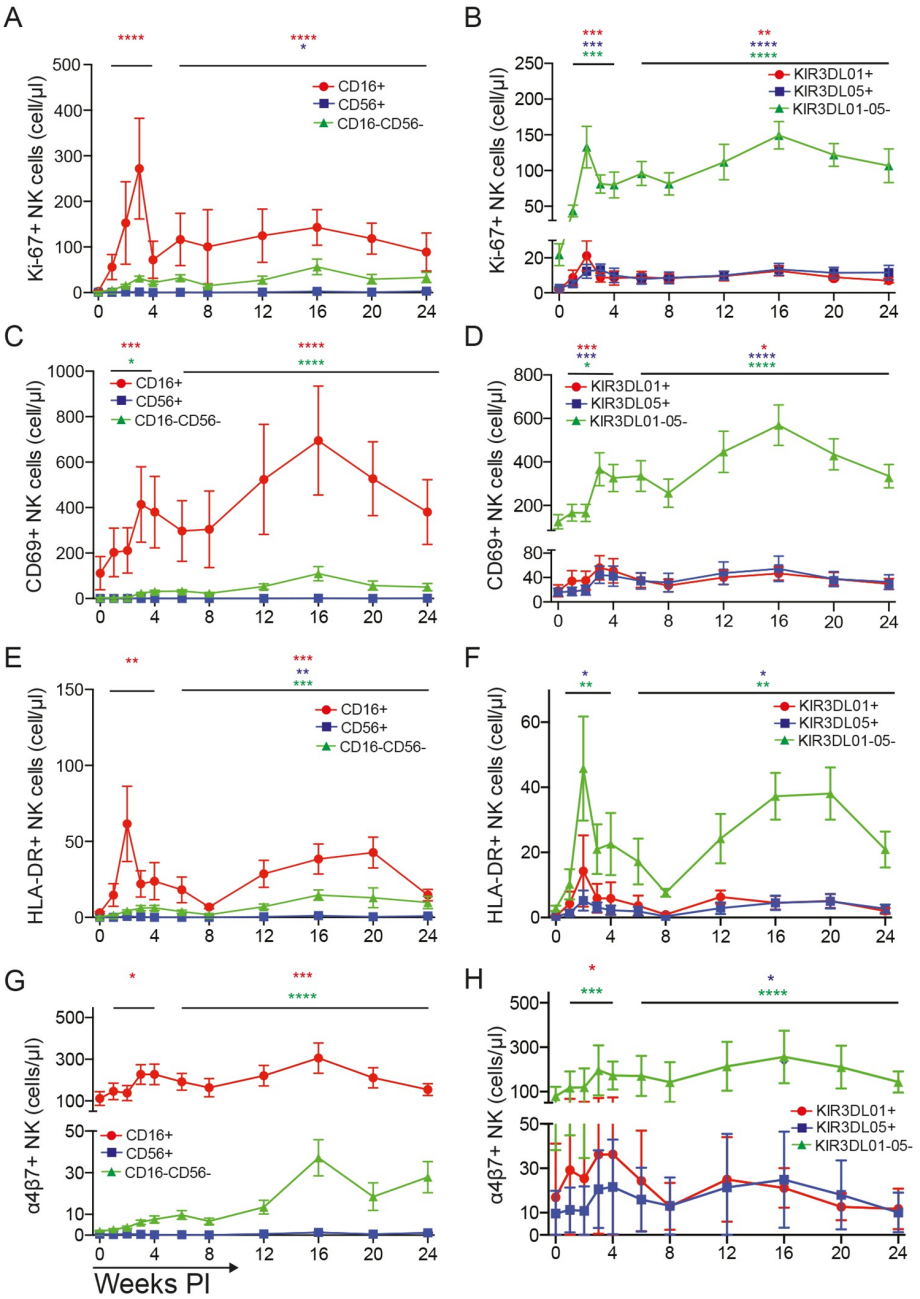
NK cell proliferation and activation in response to SIV infection. Longitudinal changes in the expression of Ki-67 (A & B), CD69 (C & D), HLA-DR (E & F), and α4β7 (G & H) were monitored for CD16^+^, CD56^+^ and CD16^-^CD56^-^ NK cells (left panels: A, C, E & G) and for KIR3DL01^+^, KIR3DL05^+^ and KIR3DL01^-^05^-^ NK cells (right panels: B, D, F & H). Absolute counts were calculated as a percentage of total NK cell counts by staining PBMCs with antibodies to CD3, CD8, NKG2A, CD16, CD56, KIR3DL01 and KIR3DL05 (tetramer), and to markers of proliferation (Ki-67), activation (CD69 & HLA-DR) and mucosal homing (α4β7). Representative gating for the proliferation and activation panel is shown in S4 Fig. The mean and standard error (error bars) are shown for each NK cell subset. Significance values for acute (weeks 1–4) and chronic (weeks 6–24) infection compared to pre-infection (week 0) are indicated with asterisks color-coded to the corresponding cell population (p<0.05*, p<0.01**, p< 0.005*** & p<0.001****, mixed effects models).

Changes were observed in the expression of proliferation and activation markers on circulating NK cells in response to SIV infection that correspond to changes in absolute NK cell counts. The percentages of NK cells expressing Ki-67 as a marker for proliferation, and CD69 or HLA-DR as activation markers, were determined for CD16^+^, CD56^+^, CD16^-^CD56^-^, KIR3DL01^+^, KIR3DL05^+^ and -KIR3DL01^-^05^-^ NK cells (S4 Fig), and used to calculate absolute counts for each subset. Since differences in NK cell counts were not observed for Mamu-A1*002^+^ versus–A1*002^-^ animals, data from all eleven KIR3DL01^+^ animals and all twelve KIR3DL05^+^ animals were analyzed together. Significant increases were detected in the number of cells expressing Ki-67, CD69 and HLA-DR during acute and chronic infection (Fig 4). The most significant increases were observed for CD16^+^ NK cells (Fig 4A, 4C and 4E), which represent the largest and most functionally mature NK cell population in peripheral blood. Increases in the number of cells expressing Ki-67 were particularly evident during acute infection (Fig 4A and 4B), indicating that changes in NK cell counts during the first three weeks of SIV infection are probably due, at least in part, to cell proliferation. Higher numbers of KIR3DL01^+^, KIR3DL05^+^ and KIR3DL01^-^05^-^ NK cells expressing CD69 and HLA-DR further indicate broad NK cell stimulation (Fig 4D and 4F). Thus, changes in NK cell activation and proliferation parallel increases in cell numbers during acute and chronic SIV infection.

To assess the potential of NK cells to home to tissues, PBMCs were stained with a panel that included antibodies to the mucosal homing receptor α4β7 as an indicator of trafficking to the intestinal mucosa, the chemokine receptor CCR7 as a marker for trafficking to peripheral lymphoid tissues and the chemokine receptor CXCR3 as a marker for trafficking to sites of inflammation. The numbers of CD16^+^, CD56^+^, CD16^-^CD56^-^, KIR3DL01^+^, KIR3DL05^+^ and KIR3DL01^-^05^-^ NK cells expressing each of these markers were calculated from absolute counts as described above. Relatively few circulating NK cells expressed CCR7 or CXCR3, and with the exception of a modest increase in CXCR3^+^ KIR3DL01^-^05^-^ NK cells during acute infection, significant changes for these markers were not detected (S5 Fig). Significant increases were, however, observed in the frequency of CD16^+^ and CD16^-^CD56^-^ NK cells expressing α4β7, particularly during chronic infection (Fig 4G). Among the KIR-defined subsets, the KIR3DL01^-^05^-^ population exhibited the greatest increase in the percentage of α4β7^+^ cells, with significant increases in the frequency of α4β7 also detectable for KIR3DL01^+^ cells during acute infection and KIR3DL05^+^ cells during chronic infection (Fig 4H).

### NK cell degranulation and cytokine release

The potential antiviral activity of circulating NK cells was assessed by staining for functional markers of degranulation and cytokine release. PBMCs were incubated overnight, with and without stimulation with MHC class I-deficient 721.221 cells, and in the presence of an antibody to CD107a as a marker for degranulation. The cells were then stained the following day with reagents to differentiate KIR3DL01^+^ and KIR3DL05^+^ NK cells and for intracellular accumulation of TNFα (S6 Fig). Because of variations in cell viability as a result of overnight incubation, these markers were analyzed as percentages of their respective NK cell populations rather than calculating absolute cell counts.

**Fig 5:**
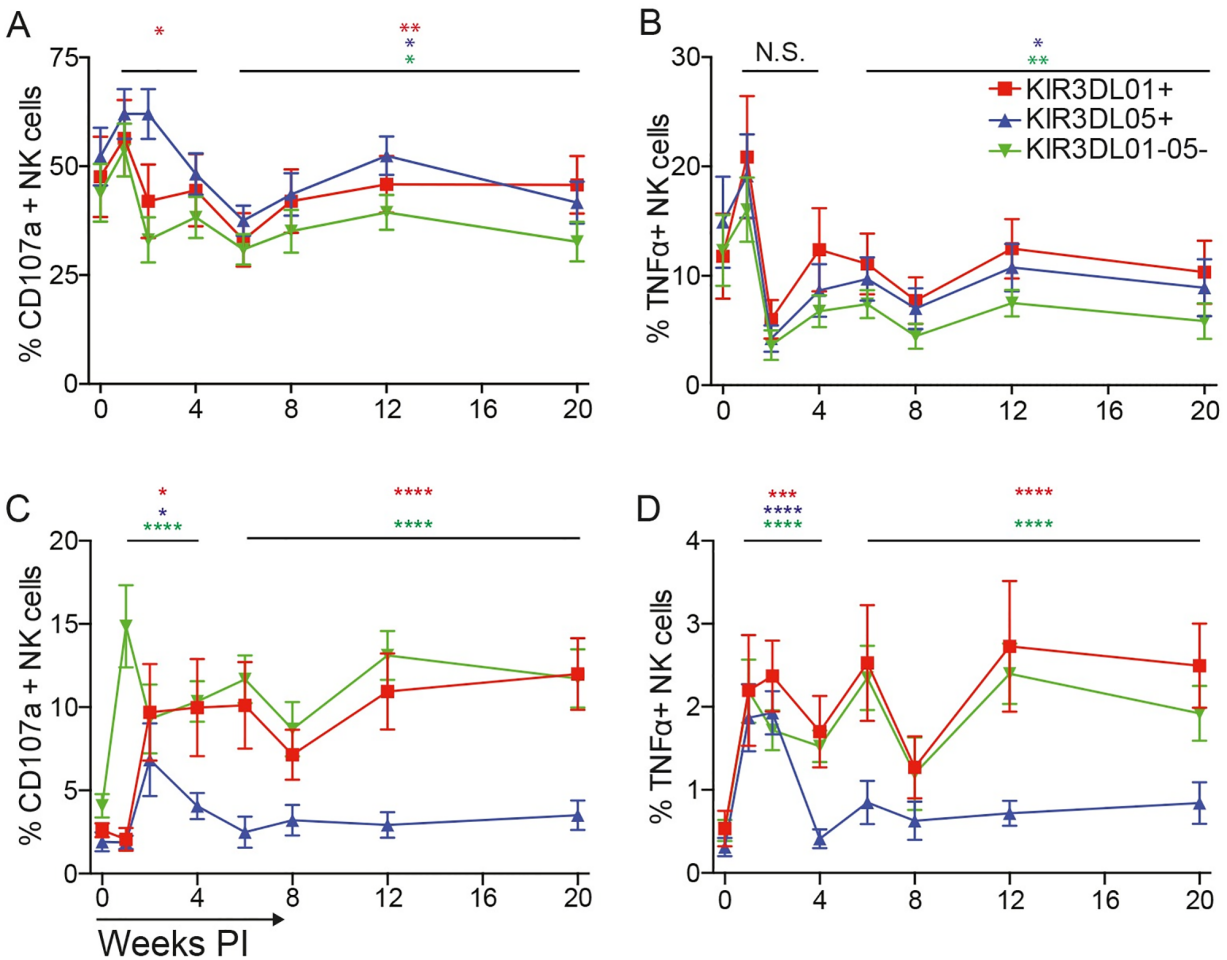
NK cell degranulation and cytokine release in response to SIV infection. Longitudinal changes in the upregulation of CD107a (A & C) and TNFα (B & D) are shown for KIR3DL01^+^, KIR3DL05^+^ and KIR3DL01^-^05^-^ NK cells in response to stimulation with 721.221 cells (A & B) or without 721.221 cell stimulation (C & D). PBMCs were incubated overnight with or without 721.221 cells in the presence of an antibody to CD107a, and stained the following day with antibodies to differentiate KIR3DL01^+^, KIR3DL05^+^ and KIR3DL01^-^05^-^ NK cells and for intracellular accumulation of TNFα. Representative gating for CD107a and TNFα staining is shown in S6 Fig. The mean and standard error (error bars) are plotted for the each NK cell population. Significance values for acute (weeks 1–4) and chronic (weeks 6–24) infection compared to pre-infection (week 0) are indicated with asterisks color-coded to the corresponding cell population (p<0.05*, p<0.01**, p< 0.005*** & p<0.001****, mixed effects models).

In accordance with the especially broad and potent “missing self” stimulus provided by the 721.221 cell line, the overall magnitude of CD107a and TNFα upregulation was much higher in response to incubation with 721.221 cells than in the absence of these cells; however, differences in the expression of these markers were not detectable among KIR3DL01^+^, KIR3DL05^+^ and KIR3DL01^-^05^-^ NK cell subsets (Fig 5A and 5B). We therefore focused on NK cell responses without 721.221 cells, occurring as a result of *in vivo* and/or *ex vivo* activation by SIV-infected cells, as a more physiological reflection of antiviral activity. In the absence of 721.221 cells, CD107a and TNFα were strongly upregulated on KIR3DL01^-^05^-^ NK cells during acute and chronic infection (Fig 5C and 5D). Although CD107a expression on KIR3DL01^+^ and KIR3DL05^+^ NK cells was delayed relative to the KIR3DL01^-^05^-^ population, significant increases in both CD107a and TNFα were also detected for these subsets during acute infection (Fig 5C and 5D); however, while these responses were sustained for KIR3DL01^+^ NK cells, the percentage of KIR3DL05^+^ NK cells expressing CD107a and TNFα declined to baseline levels during chronic infection (Fig 5C and 5D). Hence, these results reveal functional differences in degranulation and cytokine release that suggest greater antiviral activity for KIR3DL01^+^ NK cells than for KIR3DL05^+^ NK cells.

### NK cell responses in lymphoid tissues

**Fig 6:**
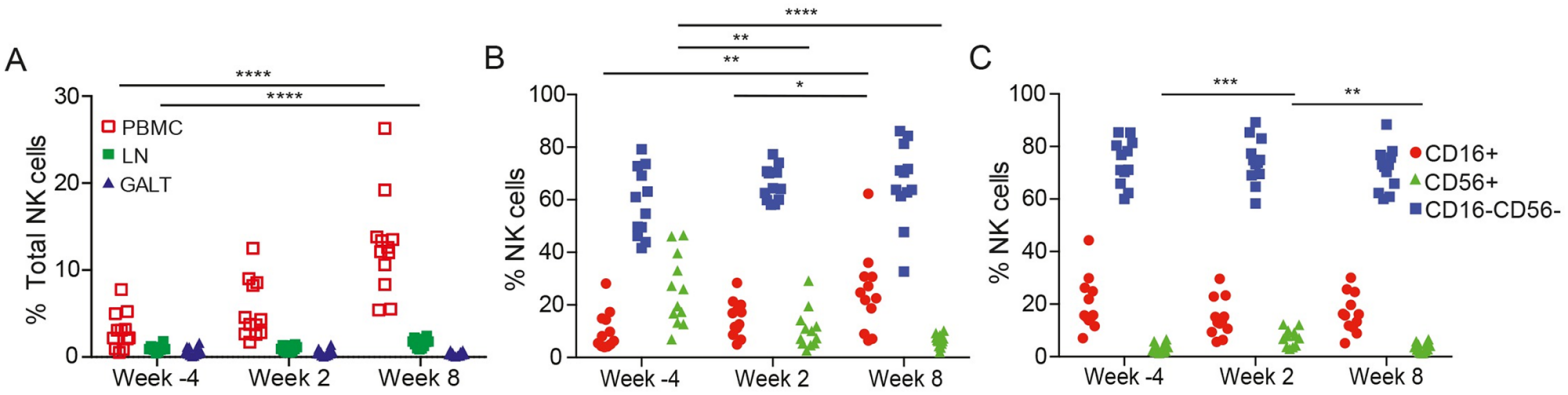
NK cell responses in tissues. The frequency of total NK cells in PBMCs, lymph nodes (LN) and gut-associated lymphoid tissues (GALT) (A), and of CD16^+^, CD56^+^ and CD16^-^CD56^-^ NK cells in LN (B) and GALT (C) during acute (week 2) and chronic (week 8) infection were compared to the frequency of these populations prior to SIV infection (week -4). Significant differences are indicated with asterisks (p<0.05*, p<0.01**, p< 0.005*** & p<0.001****, Mann-Whitney *U*-test).

Lymphocytes were isolated from lymph node and colorectal biopsies prior to SIV infection, and at two- and eight-weeks post-infection, to assess changes in the frequency of NK cells in these tissues. Compared to peripheral blood, NK cells constituted a relatively small percentage of lymphocytes in these tissue compartments. Before SIV infection, average NK cell frequencies in lymph nodes and gut-associated lymphoid tissues (GALT) were 1.2%±0.086 and 0.52%±0.065, respectively, compared to 7.0%±0.96 in PBMCs. By eight-weeks post-infection, a small but highly significant increase in the percentage of total NK cells was detectable in lymph nodes (Fig 6A). This increase was reflected by changes in the CD16^+^, CD56^+^ and CD16^-^CD56^-^ subsets. While the majority of NK cells in macaque lymph nodes are CD16^-^CD56^-^ [44], a significant increase in the frequency of CD16^+^ NK cells, and a corresponding decrease in the frequency of CD56^+^ cells, was observed in response to SIV infection (Fig 6B). In contrast, the percentage of total NK cells in GALT did not change following SIV infection (Fig 6A), and aside from a transient increase in the CD56^+^ population during acute infection, the proportions of CD16^+^, CD56^+^ and CD16^-^CD56^-^ cells also remained unchanged (Fig 6C).

**Fig 7:**
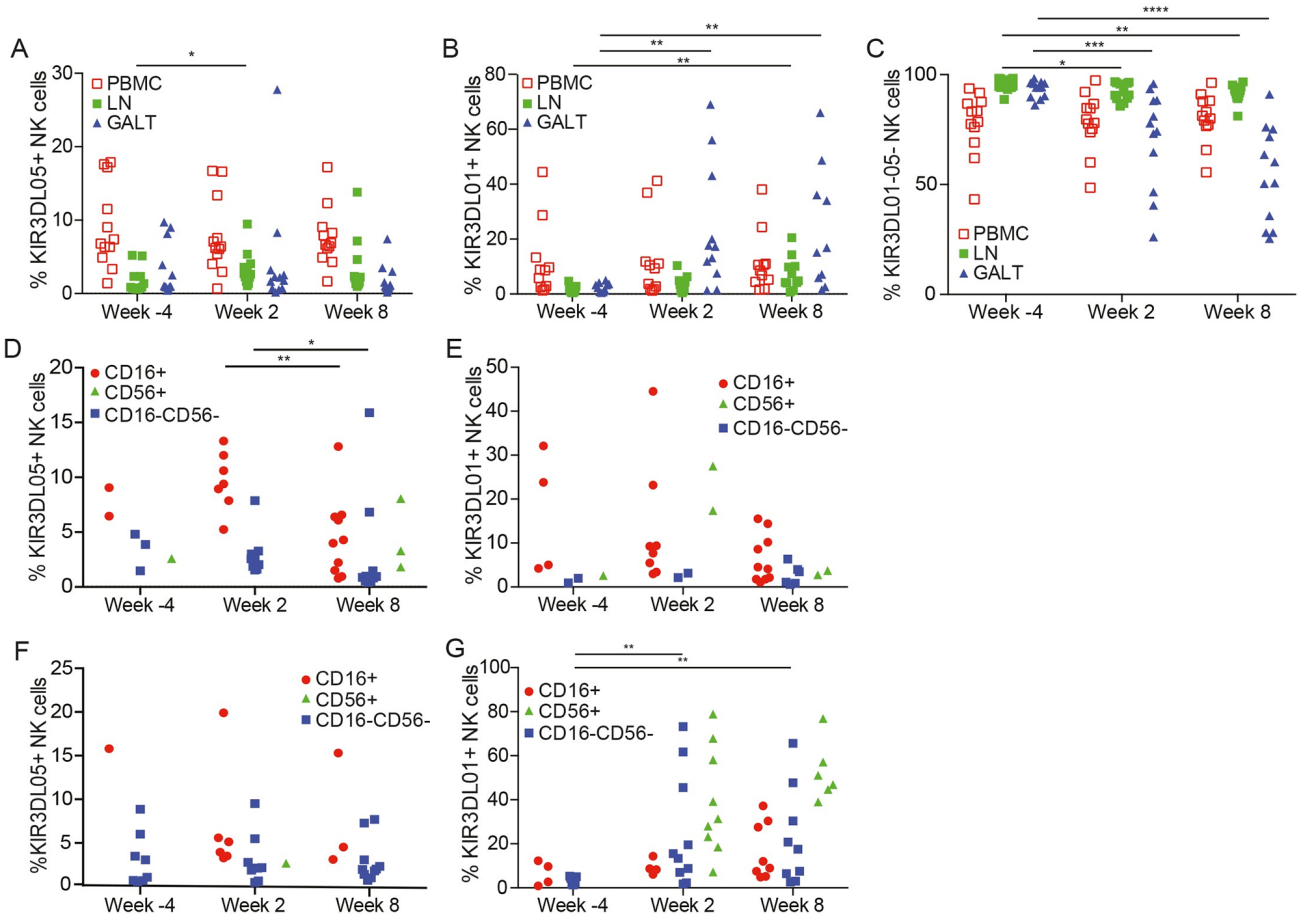
Comparison of the frequencies of KIR3DL01^+^ and KIR3DL05^+^ NK cells in lymph nodes and gut-associated lymphoid tissues. The frequencies of KIR3DL05^+^ (A), KIR3DL01^+^ (B) and KIR3DL01^-^05^-^ (C) NK cells in PBMCs, lymph nodes (LN) and gut-associated lymphoid tissues (GALT) were compared prior to SIV infection (week -4) and at weeks 2 and 8 after SIV infection. The frequencies of CD16^+^, CD56^+^ and CD16^-^CD56^-^ NK cells in lymph nodes (D & E) and gut-associated lymphoid tissues (F & G) expressing KIR3DL05 (D & F) or KIR3DL01 (E & G) were also compared at weeks -4, 2 and 8 pre- and post-infection. Representative gating for differentiating KIR3DL01^+^ versus KIR3DL05^+^ NK cells in lymphocytes isolated from colorectal biopsies is illustrated in S7 Fig. Significant differences are indicated with asterisks (p<0.05*, p<0.01**, p< 0.005*** & p<0.001****, Mann-Whitney *U*-test). Samples with less than 30 events per gate were excluded from the analysis.

**Fig 8:**
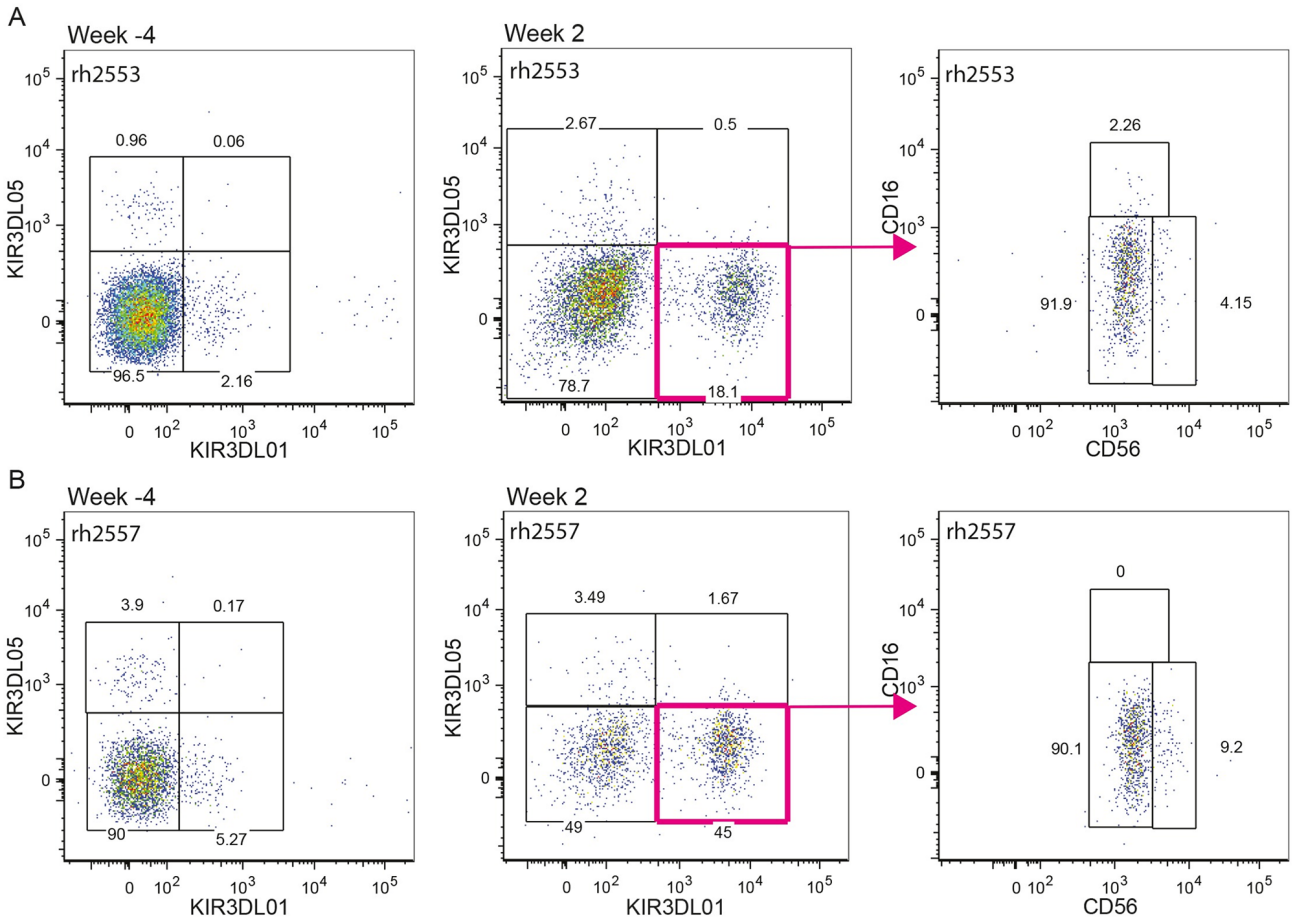
KIR3DL01 upregulation on NK cells of the gastrointestinal mucosa. Representative KIR3DL01 versus KIR3DL05 staining on NK cells isolated from colorectal biopsies is shown for two different rhesus macaques before (week -4) and after (week 2) SIV infection. CD16 versus CD56 staining is shown for the KIR3DL01^+^ population at week 2 post-infection (right panels).

To assess changes in the frequency of NK cells expressing KIR3DL01 and KIR3DL05, the percentages of these cells were compared in lymph node and colorectal biopsies. Whereas SIV infection did not alter the frequency of KIR3DL05^+^ NK cells (Fig 7A), significant increases in the frequency of KIR3DL01^+^ NK cells were observed in GALT during acute and chronic infection (weeks 2 and 8) and in lymph nodes during chronic infection (week 8) (Fig 7B), which were mirrored by corresponding reductions in the frequency of KIR3DL01^-^05^-^ NK cells in these tissues (Fig 7C). Further analysis of KIR expression revealed that, similar to peripheral blood, KIR3DL01 and KIR3DL05 are expressed by a higher percentage of CD16^+^ NK cells than CD56^+^ or CD16^-^CD56^-^ NK cells in lymph nodes (Fig 7D and 7E); however, the increased frequency of KIR3DL01^+^ NK cells appeared to reflect the upregulation of this receptor on CD56^+^ and CD16^-^CD56^-^ NK cells (Fig 7E). In the GALT, increases in the expression of KIR3DL01 (Fig 7G), but not KIR3DL05 (Fig 7F), on CD56^+^ and CD16^-^CD56^-^ NK cells were more dramatic. Indeed, increases in the expression of KIR3DL01 on CD56^+^ and CD16^-^CD56^-^ NK cells account almost entirely for the higher frequency of cells expressing this KIR after SIV infection (39.2%±7.6 and 24.9%±8.2, respectively) (Fig 7G). Representative data showing the preferential upregulation of KIR3DL01, but not KIR3DL05, on NK cells of the GALT at week 2 post-infection, and that the majority of these KIR3DL01^+^ cells are either CD56^+^ or CD16^-^CD56^-^, is provided in Fig 8.

## Discussion

KIR and HLA class I polymorphisms play a central role in the regulation of NK cell responses and can have a significant impact on the course of HIV-1 infection [17,19,23]. Studies to address the functional significance of KIR-MHC class I interactions in SIV-infected macaques have, however, been hampered by the lack of defined ligands for KIRs in non-human primates. To address this limitation, we and others have identified ligands for macaque KIRs [29,30,40,42,52]. We identified Mamu-A1*002, a common MHC class I molecule expressed in approximately 20% of Indian-origin rhesus macaques, as a ligand for KIR3DL05 [30], and found that nearly a third of the SIV peptides bound by Mamu-A1*002 suppress the cytolytic activity of KIR3DL05^+^ NK cells [16]. We further identified MHC class I molecules with a Bw4 motif as ligands for KIR3DL01, which is the most polymorphic and commonly expressed KIR in rhesus macaques [29]. To determine how these receptor-ligand interactions influence NK cell responses and the ability to contain virus replication, we infected twelve KIR- and MHC class I-defined rhesus macaques with SIV and monitored NK cell responses and viral loads in peripheral blood and lymphoid tissues.

As in humans, macaque KIRs are expressed in a variegated and stochastic fashion, which accounts for the presence of KIR3DL01 and KIR3DL05 on a fraction of total NK cells [29,30]. Unlike humans, however, which only express KIRs on functionally mature CD16^+^CD56^dim^ NK cells, KIRs are expressed on all peripheral blood NK cell subsets in macaques [47]. KIR3DL01 and KIR3DL05 were accordingly detected on CD16^+^, CD56^+^ and CD16^-^CD56^-^ NK cells. Nevertheless, these KIRs are expressed on a higher percentage of CD16^+^ cells than CD56^+^ or CD16^-^CD56^-^ cells, further supporting a functional correspondence between the CD16^+^ NK population in macaques and the dominant CD16^+^CD56^dim^ population in human blood.

The education of human NK cells is associated with an increase in the frequency of cells bearing KIRs that recognize self HLA class I ligands and a corresponding decrease in surface staining for those receptors [48–50]. Although neither the frequency of KIR3DL01^+^ or KIR3DL05^+^ NK cells, nor the intensity of KIR3DL01 staining, was associated with differences the number of MHC class I alleles predicted to encode ligands for these receptors, the level of KIR3DL05 staining correlated inversely with the presence of Mamu-A1*002 and -A3*13. This correlation appears to be driven primarily by Mamu-A1*002, since independent comparisons revealed significant differences in KIR3DL05 staining for Mamu-A1*002^+^ versus -A1*002^-^ animals, but not for Mamu-A3*13^+^ versus -A3*13^-^ animals; however, since KIR3DL05 staining was lower for Mamu-A3*13^+^ animals than for animals lacking both Mamu-A1*002 and -A3*13, and most of the Mamu-A1*002^+^ animals were also -A3*13^+^ as a consequence of linkage disequilibrium between these alleles [53], it is possible that Mamu-A3*13 may have a modest influence on KIR3DL05 levels that could be additive in combination with Mamu-A1*002. Nevertheless, a more dominant effect of Mamu-A1*002 on KIR3DL05 staining would be consistent with the unusually high avidity of Mamu-A1*002 for KIR3DL05 [30] and with higher levels of Mamu-A1*002 expression than for gene products of the ‘minor’ Mamu-A3*13 locus [51]. Thus, lower levels of KIR3DL05 staining for Mamu-A1*002^+^ macaques suggest a pronounced effect of Mamu-A1*002 on the education of KIR3DL05^+^ NK cells.

It should be noted, however, that the nature of NK cell education, or licensing, is not fully understood. It is possible that reduced levels of KIR3DL05 on NK cells from Mamu-A1*002^+^ animals reflect the binding of Mamu-A1*002 to KIR3DL05 on the same cells, similar to *cis* interactions previously described for MHC class I ligands of murine Ly49A [54] and human leukocyte Ig-like receptors (LILRs) [55,56]. Although to our knowledge *cis* interactions have not been reported for KIRs, it is tempting to speculate that high avidity interactions between Mamu-A1*002 and KIR3DL05 may sequester KIR3DL05, thereby reducing the accessibility of this receptor for staining on the cell surface. Such interactions would not necessarily be inconsistent with the functional effects of KIR ligands on NK cell licensing, since the sequestration of inhibitory KIRs may reduce the threshold required for NK cell activation through these receptors.

Although SIV peptides bound by Mamu-A1*002 were recently shown to suppress the cytolytic activity of Mamu-KIR3DL05^+^ NK cells, neither viral loads nor NK cell responses differed for Mamu-A1*002^+^ versus–A1*002^-^ animals. Hence, these results did not reveal an advantage to SIV replication in Mamu-A1*002^+^ animals as a result of the presentation of inhibitory peptides to KIR3DL05^+^ NK cells, as *in vitro* assays with sorted primary NK cells might suggest [16]. However, these findings do not necessarily preclude a contribution of peptides to the evasion of NK cell responses. Rhesus macaques typically express six to thirteen different KIRs [36,40], and the ligands for most of these receptors remain undefined. Macaque NK cells also express other more conserved inhibitory and activating receptors, such as CD94/NKG2 heterodimers and natural cytotoxicity receptors (NCRs) that may influence responses to viral infection. Thus, it is possible that the effects of Mamu-A1*002-bound viral peptides on KIR3DL05^+^ NK cells may have been obscured by other receptor-ligand interactions. Furthermore, while lower levels of KIR3DL05 on NK cells from Mamu-A1*002^+^ animals suggest a dominant effect of Mamu-A1*002 on the education of KIR3DL05^+^ NK cells, other MHC class I molecules may also serve as ligands for this receptor. Therefore, given the complexity of *KIR* and *MHC class I* immunogenetics in rhesus macaques, and our limited knowledge of receptor-ligand interactions in this species, it is perhaps not surprising that we did not detect gross differences in viral loads or NK cell responses as a result of peptide-dependent modulation of a single KIR ligand.

The onset of adaptive immunity may also have complicated NK cell responses during chronic infection. Since previous studies have shown that the selection of CD8^+^ T cell escape variants in most of the SIV epitopes bound by Mamu-A1*002 generally occurs only after months of chronic infection [57–59], CD8^+^ T cell escape is unlikely to have affected KIR3DL05^+^ NK cell responses or viral loads during acute infection; however, we cannot exclude the possibility that the emerge of escape variants may have contributed to variability in KIR3DL05^+^ NK responses during chronic infection.

SIV infection nevertheless broadly stimulated NK cell responses. Within the first four weeks of infection, rapid increases in total NK cell counts, as well as cell counts for the CD16^+^ and CD16^-^CD56^-^ populations, were observed in peripheral blood. In accordance with cross-sectional comparisons, these increases were maintained during chronic infection [44]. Similar changes were observed in NK cell populations defined by KIR3DL01 and KIR3DL05. Increases in KIR3DL01^+^, KIR3DL05^+^ and KIR3DL01^+^05^+^ NK cells during acute infection paralleled increases in total NK cells counts; however, while the number of KIR3DL05^+^ NK cells remained elevated during chronic infection, these increases were not sustained for the KIR3DL01^+^ subset. Longitudinal analyses revealed additional differences in the percentage of CD16^+^ NK cells in blood expressing KIR3DL01 versus KIR3DL05. Whereas the percentage of CD16^+^ NK cells expressing KIR3DL05 was relatively unchanged, there was a gradual decrease in the frequency of KIR3DL01^+^ CD16^+^ NK cells after eight weeks of infection. Although this decrease was partially offset by increases in the frequency of CD56^+^ and CD16^-^CD56^-^ cells expressing this KIR, the decline of the larger KIR3DL01^+^CD16^+^ population probably accounts for lower overall KIR3DL01^+^ NK cell counts in blood during chronic infection.

Phenotypic analyses further revealed rapid increases in NK cell activation in response to SIV infection. Consistent with increases in circulating NK cell counts, the proliferation marker Ki-67 was highly upregulated during acute and chronic infection on KIR3DL01^+^, KIR3DL05^+^ and KIR3DL01^-^05^-^ NK cells. Similar increases were also observed in the number of NK cells expressing the activation markers CD69 and HLA-DR, and the mucosal homing receptor α4β7. Markers of antiviral activity, including CD107a and TNFα, were likewise broadly upregulated during acute infection; however, the expression of these markers on KIR3DL01^+^ versus KIR3DL05^+^ NK cells diverged during chronic infection. Whereas CD107a and TNFα continued to be upregulated on KIR3DL01^+^ and KIR3DL01^-^05^-^ cells, the percentage of KIR3DL05^+^ NK cells expressing these antigens declined to baseline levels by week six post-infection. These results reveal differential antiviral responses for KIR3DL05^+^ versus KIR3DL01^+^ NK cells, perhaps reflecting a greater role for the KIR3DL01^+^ subset in controlling SIV replication.

Characterization of NK cell subsets in lymph nodes and the gastrointestinal mucosa revealed additional changes in response to SIV infection. Although NK cells constitute a relatively minor percentage of lymphocytes in these tissues, a highly significant increase in the frequency of total NK cells was detected in lymph nodes during chronic infection. Consistent with previous studies [60], we found that the majority of NK cells in lymph nodes are CD16^-^CD56^-^, and of the smaller populations of CD16^+^ and CD56^+^ NK cells, the CD56^+^ subset is more prevalent in naïve animals. Following SIV infection, however, there was an inversion in the frequency of CD16^+^ versus CD56^+^ NK cells, with an accumulation of CD16^+^ NK cells and a corresponding decrease in the percentage of CD56^+^ NK cells. Significant increases were also detected in lymph node frequencies of KIR3DL05^+^ and KIR3DL01^+^ NK cells during acute and chronic infection, respectively.

In gut-associated lymphoid tissues, the percentage of total NK cells did not change in response to SIV infection, and with the exception of a transient increase in the frequency of the CD56^+^ subset, the relative proportions of CD16^+^, CD56^+^ and CD16^-^CD56^-^ NK cells in this compartment also remained unchanged. However, dramatic changes were observed in the frequency of KIR3DL01^+^ NK cells. Significant increases in the percentage of KIR3DL01^+^ NK cells in GALT were detected during both acute and chronic infection. Surprisingly, most of these cells were CD56^+^ or CD16^-^CD56^-^, suggesting that this increase reflects the upregulation of KIR3DL01 on less mature NK cells rather than the accumulation of CD16^+^ NK cells expressing this KIR. Moreover, this response appears to be specific to KIR3DL01, since similar increases were not observed for KIR3DL05^+^ cells.

The enrichment of KIR3DL01^+^ NK cells in the gastrointestinal mucosa is intriguing, since the gut-associated lymphoid tissues are known to be a major source of HIV-1 and SIV replication and CD4^+^ T cell turnover [61–64]. Although rhesus KIR3DL01 and human KIR3DL1 are not orthologous, they may have similar functions; both recognize Bw4 ligands and are the most polymorphic KIRs of their respective species [29]. Moreover, the lysis of HIV-infected cells by KIR3DL1^+^ NK cells is primarily triggered by downmodulation of HLA-Bw4 ligands from the cell surface by the viral Nef protein [8,22], and we previously demonstrated that Mamu-Bw4 molecules are efficiently downmodulated by SIV Nef [65]. Thus, increases in the frequency of KIR3DL01^+^ NK cells in the GALT may reflect an innate cellular response to especially high levels of SIV replication and Bw4 downmodulation in these tissues.

To our knowledge, this study represents the first longitudinal analysis of NK cell responses in *KIR*- and *MHC class I*-defined macaques. Responses to SIV infection in peripheral blood were characterized by rapid increases in the CD16^+^ and CD16^-^CD56^-^ populations, including KIR3DL01^+^ and KIR3DL05^+^ subsets. Markers of proliferation, activation and antiviral activity were widely expressed during acute infection, but began to diverge after four weeks, as indicated by sustained CD107a and TNFα upregulation by KIR3DL01^+^ NK cells, but not by KIR3DL05^+^ NK cells. Differential responses for KIR3DL01^+^ versus KIR3DL05^+^ NK cells were also evident in tissues. Whereas the percentages of KIR3DL05^+^ NK cells in lymph nodes and the gastrointestinal mucosa did not change, significant increases were observed in the frequency of KIR3DL01^+^ NK cells, especially in gut-associated lymphoid tissues. Thus, our results reveal broad NK cell activation and dynamic changes in multiple subsets in response to SIV infection, including an enrichment of KIR3DL01^+^ NK cells in mucosal tissues that represent major sites of ongoing virus replication and CD4^+^ lymphocyte depletion.

## Materials and methods

### Ethics statement

Twelve rhesus macaques (*Macaca mulatta*) of Indian origin, including six male and six female animals, were used in this study. Housing and care of the animals at the Wisconsin National Primate Research Center (WNPRC) were in compliance with the standards of the American Association for the Accreditation of Laboratory Animal Care and the University of Wisconsin Research Animal Resources Center (UWRARC). Animal experiments were approved by the UWRARC (protocol number G005496) and conducted in accordance with the principles described in the *Guide for the Care and Use of Laboratory Animals* [66]. Steps to improve animal welfare included environmental enrichment, such as foraging opportunities and manipulatable devices. Water was continuously available, commercial monkey chow was provided twice daily and fresh produce was supplied three times per week. Animals were sedated with ketamine HCl prior to the collection of blood and biopsy samples to minimize pain and distress associated with experimental procedures and were monitored twice daily by animal care and veterinary staff.

### KIR- and MHC class I-genotyping

Twelve *KIR3DL05*^+^ rhesus macaques, including six Mamu-A1*002^+^ and six–A1*002^-^ animals, were selected for this study. These included eleven animals expressing allotypes of KIR3DL01 with aspartic acid at position 233 (KIR3DL01 D233) [29] and excluded animals expressing the MHC class I alleles Mamu-A1*001, -B*008 and–B*17 associated with spontaneous control of SIV replication [58,67–69]. KIR3DL05^+^ and KIR3DL01 D233^+^ macaques were identified by staining PBMCs with Mamu-A1*002 Gag GY9 tetramer and the NKVFS1 monoclonal antibody as previously described [29,30]. All animals were also genotyped by sequencing full-length *KIR* transcripts as recently described [70]. Briefly, RNA was isolated from PBMCs, and full-length *KIR* cDNA was sequenced using a PacBio RS II instrument with P6-C4 sequencing reagents. Sequences were identified and novel alleles were classified by comparison to previously reported alleles of rhesus macaque *KIRs*. GenBank accession numbers for newly identified *KIR* alleles are listed in S1 Table. MHC class I genotyping was performed using genomic DNA isolated from PBMC by sequencing a 150 bp region of exon 2 (Illumina MiSeq system). Sequences were analyzed by comparison to an in-house database as previously described [71]. The *MHC class I-* and *KIR-*genotypes of each of the animals in this study are summarized in Tables 1 and 2.

### SIV_mac_239 infection

Animals were infected intravenously with SIV_mac_239. A vial of SIV_mac_239 challenge stock prepared in activated rhesus macaque PBMC was provided by Dr. Ronald Desrosiers, Miller School of Medicine, University of Miami. On the day of inoculation, the vial was thawed and diluted to 50 animal infectious doses per ml in sterile, serum-free RPMI. Within 30 minutes of preparation, a one ml dose of the virus dilution (7.8 pg p27) was administered to each animal under ketamine anesthesia through a 22 g catheter placed aseptically in the saphenous vein.

### Plasma viral RNA load measurements

Plasma was collected from blood drawn in tubes with EDTA as an anticoagulant and cryopreserved at -80°C. Virus was pelleted from 0.5 to 1.0 ml of plasma by ultracentrifugation for one hour at 20,000 x g. Viral RNA was extracted, reverse transcribed into cDNA, and quantified by real-time PCR using an assay based on amplification of a conserved SIV *gag* sequence as previously described [72].

### Lymphocyte isolation

#### i. Peripheral blood

Peripheral blood was collected in tubes containing EDTA as an anticoagulant. Plasma was separated from whole blood by centrifugation at 1680 x g and stored at -80°C for viral RNA load measurements. The blood was then diluted with two volumes of sterile PBS, layered over Ficoll Paque PLUS (GE Healthcare) and centrifuged for 30 minutes at 830 x g. PBMCs were collected from the gradient interface, washed twice in PBS 10% FBS, and yields were determined using a Countess Automated Cell Counter (LifeTechnologies). For live cell counts, the PBMC were treated with Turk’s solution (0.5% acetic acid in Trypan blue) for erythrocyte lysis and live/dead discrimination.

#### ii. Lymph nodes

Axillary and inguinal lymph nodes were collected from macaques by excisional biopsy. Lymphocytes were released from lymph nodes by mechanical disruption in a 100 mm dishes, collected in sterile PBS and passed through a 100 μm nylon mesh. Lymphocytes were then separated over Ficoll Paque PLUS gradients (30 min, 830 x g), washed twice in PBS 10% FBS, and either used for flow cytometry or cryopreserved in liquid nitrogen.

#### iii. Gut-associated lymphoid tissue

Lymphocytes were isolated from biopsies of the colon and rectum as previously described [61,73] to monitor the homing of NK cells to the gastrointestinal mucosa. Pinch biopsies of the epithelium and underlying lamina propria were collected from 10–12 sites in the colon and rectum and pooled to maximize cell yield. The samples were incubated at 37°C with vigorous shaking in HBSS supplemented with 1 mM EDTA for 30 minutes (ThermoFisher), followed by one hour in 0.5 mg/ml type II collagenase (Sigma-Aldrich). The tissues were then mechanically dispersed by repeated pipetting, filtered through a 100 μm nylon mesh and pelleted by centrifugation. Cells were then resuspended in 35% Percoll and layered over 60% discontiguous Percoll (Sigma-Aldrich) gradients and centrifuged at 830 x g for 30 minutes at 4°C with slow acceleration and brake off. Lymphocytes were collected from the interface between the 35% and 60% Percoll layers, washed, and suspended in PBS with 10% FBS for flow cytometry.

### Flow cytometry

Unless specified otherwise, all antibodies were purchased from BD Biosciences. All flow cytometry data were collected using a BD LSRII SORP and analyses were done with FlowJo 9.9 software (TreeStar Inc.).

#### i. Absolute counts

Whole blood (50 μl) was stained for 15 minutes at room temperature in Trucount tubes (BD Biosciences) with anti-CD8 PE (Clone SK1), anti-CD4 BV510 (clone L200), anti-CD45 PerCP-Cy5.5 (clone D058-1283), anti-CD3 PE-CF594 (clone Sp34-2), anti-CD20 Alexa700 (clone 2H7), anti-CD159a APC (Clone z199, Beckman Coulter) and anti-HLA-DR APC-Cy7 (clone L243). The samples were then treated for 15 minutes at room temperature with 450 μl BD FACS lysing solution to lyse erythrocytes and fix mononuclear cells. The cells were analyzed by flow cytometry, collecting at least 5000 fluorescent bead events. Absolute counts for NK cells (CD45^+^CD3^-^CD20^-^CD8^+^), B cells (CD45^+^CD20^+^), CD4^+^ T cells (CD45^+^CD3^+^CD4^+^) and CD8^+^ (CD45^+^CD3^+^CD8^+^) T cells were determined by dividing the number of gated cellular events by the number of bead events and multiplying by the bead concentration.

#### ii. Proliferation and activation panel

PBMCs (2x10^6^ cells) or lymph node mononuclear cells (5x10^6^ cells) were stained for one hour at 37°C with BV421-conjugated Mamu-A1*002 Gag_71-79_ GY9 tetramer (NIH Tetramer core facility, Emory University Vaccine Center), followed by 15 minutes at room temperature with anti-CD8 BV510 (clone SK1), anti-CD16 BV711 (clone 3G8), anti-HLA-DR BV650 (clone L243, BioLegend), anti-CD45 BV786 (clone D058-1283), anti-CD56 FITC (clone B159), anti-KIR2D PE (clone NKVFS1, Miltenyi Biotec), anti-CD69 ECD (clone TP1.55.3, Beckman Coulter), anti-NKp46 PE-Cy5 (clone BAB281, Beckman Coulter), anti-NKG2A (CD159a) PE-Cy7 (clone z199, Beckman Coulter), anti-CD3 Alexa700 (clone SP34-2), anti-CD14 Alexa700 (M5E2), anti-CD20 Alexa700 (clone 2H7) and NearIR live/dead cell stain (Invitrogen). Samples were also stained with isotype controls for anti-HLA-DR BV650 (clone MOPC-174, BioLegend), anti-CD69 ECD (MPC-11) and anti-NKp46 PE-Cy5 (clone 2T8-2F5, Beckman Coulter). The cells were then fixed in 2% paraformaldehyde (PFA) PBS, permeabilized with 0.1% saponin (Thermo Fisher Scientific) PBS 10% FBS, and stained for 50 minutes with anti-Ki-67 Alexa647 (clone B56) or an isotype control antibody (MOPC-21). Following intracellular staining, the cells were washed and resuspended in 2% PFA PBS for flow cytometry. The percentages of KIR3DL01^+^ versus KIR3DL05^+^ NK cells and CD16^+^ versus CD56^+^ NK cells expressing Ki-67, CD69 and HLA-DR were determined after gating on CD45^+^CD3^-^CD14^-^CD20^-^CD8^+^ lymphocytes. Absolute counts for these populations were calculated as a percentage of total NK cells from Trucount analysis of whole blood as described above.

#### iii. Homing panel

PBMCs (2x10^6^ cells) were stained for one hour at 37°C with BV421-conjugated Mamu-A1*002 Gag_71-79_ GY9 tetramer, followed by 15 minutes at room temperature with anti-KIR2D PE, anti-CCR7 PE-CF594 (clone 150503), anti-CD56 PerCp-Cy5.5 (clone B159), anti-NKG2A PE-Cy7 (clone z199), anti-CD8 BV510 (clone SK1), anti-CXCR3 BV711 (clone 1c6/CXCR3), anti-CD16 BV786 (clone 3G8), anti- α4β7 APC (clone A4B7, NIH Nonhuman Primate Reagent Resource), anti-CD3 Alexa700 (clone SP34-2), anti-CD14 Alexa700 (clone M5E2), anti-CD20 Alexa700 (clone 2H7) and NearIR live/dead stain. For lymph node samples, CD45-BV605 (clone D058-1283) was included. Isotype controls for anti-CXCR3 BV711 (clone X40), anti-CCR7 PE-CF594 (G155-178) anti-α4β7 APC (MOPC-21) were also included. The cells were then fixed in 2% PFA PBS, permeabilized with 0.1% saponin PBS 10% FBS, and stained for 50 minutes with anti-Perforin FITC (clone 344 MabTech) or an isotype control (clone J606, BD Pharmingen). After intracellular staining, the cells were washed and resuspended in 2% PFA PBS for flow cytometry. The percentages of KIR3DL01^+^ versus KIR3DL05^+^ NK cells expressing α4β7, CCR7 and CXCR3 were determined after gating on CD3^-^CD14^-^CD20^-^CD8^+^ lymphocytes. Absolute counts for these populations were calculated as a percentage of total NK cells from Trucount analysis of whole blood as described above.

#### iv. Degranulation and cytokine release panel

PBMCs (2x10^6^ cells) were incubated in the presence or absence of 8x10^5^ 721.221 cells (2.5:1 E:T ratio) for 16 hours at 37°C in R10 medium (RPMI supplemented with 10% FBS, L-glutamine and Primocin) with anti-CD107a BV605 (Clone H4A3, BioLegend), 5 μg/ml Brefeldin A (BioLegend) and 1 μM Monensin (BioLegend). The cells were then stained for 1 hour at 37°C with Mamu-A1*002 Gag_71-79_ GY9 APC tetramer, followed by 20 minutes at room temperature with anti-KIR2D PE (Clone NKVFS1), anti-CD3 PE-CF594 (Clone SP34-2), anti-CD56 PerCp-Cy5.5 (clone B159), anti-CD159a PE-Cy7 (Clone z199), anti-CD16 Pacific Blue (clone 3G8), anti-CD8 BV711 (clone SK1) and NearIR live/dead cell stain. Following fixation in 2% PFA PBS and permeabilized with 0.1% saponin PBS 10% FBS, the cells stained for 50 minutes with anti-IFNγ FITC (clone 4S.B3), anti-Granzyme B BV510 (clone GB11), anti-TNFα Alexa700 (clone Mab11) or an isotype control for IFNγ FITC (clone MOPC-21). After intracellular staining, the cells were washed and resuspended in 2% PFA PBS for flow cytometry. The percentages of KIR3DL01^+^ versus KIR3DL05^+^ NK cells expressing CD107a, IFNγ and TNFα were determined after gating on CD3^-^CD8^+^NKG2A^+^ lymphocytes.

### Statistical analysis

A linear mixed-effect model was used for the analysis of longitudinal data. An individual macaque was included as a random-effect to account for correlation within subjects. The presence or absence of Mamu-A1*002 and the time points after infection were included as fixed-effects in the models. The three phases of the infection were naïve or pre-infection (week 0), acute infection (weeks 1–4) and chronic infection (weeks 5–24). Two-sided p-values less than 0.05 were considered statistically significant. For the comparison of discrete data, Mann-Whitney U tests were performed using GraphPad Prism V6g.

## Supporting information

S1 Fig Gating to determine absolute counts for lymphocyte subsets. Whole blood was stained with antibodies to CD45, CD3, CD4, CD8, CD20, NKG2A (CD159a) and HLA-DR. CD45^+^ cells were subdivided into CD20^+^, CD3^+^ or CD20^-^CD3^-^ lymphocytes. The CD3^+^ lymphocytes were further subdivided in CD4^+^ and CD8^+^ T cell subsets. NK cells were defined as CD45^+^CD3^-^CD8^+^ lymphocytes and further confirmed as NKG2A^+^. Gating for enumeration of the Trucount beads is shown in the bottom panels.
(PDF)

S2 Fig Longitudinal changes in mean NK and T cell counts in peripheral blood in response to SIV infection. The means and standard error (error bars) of absolute counts for total NK cells (A), CD8^+^ T cells (B), CD4^+^ T cells (C), KIR3DL01^+^ NK cells (D), KIR3DL05^+^ NK cells (E), KIR3DL01^-^05^-^ NK cells (F), KIR3DL01^+^05^+^ NK cells (G), CD16^+^ NK cells (H), CD56^+^ NK cells (I) and CD16^-^CD56^-^ NK cells (J) are shown for Mamu-A1*002^+^ versus–A1*002^-^ animals. Gating strategies for determining absolute lymphocyte counts in blood and the percentages of PBMCs expressing CD16, CD56, KIR3DL01 and KIR3DL05 are shown in S1 and S4 Figs. Statistics were calculated using a mixed effects model by comparing results from acute (week 1–4) and chronic (weeks 6–24) infection to pre-infection (week 0) (p<0.05 *, p<0.01**, p< 0.005*** & p<0.001****).
(PDF)

S3 Fig KIR staining as a function of Mamu-A3*13 and–Bw4 alleles. Comparison of the mean fluorescence intensity of KIR3DL05 staining on NK cells from Mamu-A13*13^+^ (blue) versus Mamu-A3*13^-^ (red) animals prior to SIV infection (week 0) and at weeks 2, 8 and 20 post-infection (A). Differences in KIR3DL05 staining were not significant (N.S.) by Mann-Whitney *U*-test comparisons. Comparison of the mean fluorescence intensity of KIR3DL01 staining on NK cells versus the number of alleles predicted to encode Mamu-Bw4 ligands for this receptor prior to SIV infection (week 0) and at weeks 2, 8 and 20 post-infection (B). Linear regression analysis did not reveal significant correlations between KIR3DL01 staining and the number of Mamu-Bw4 alleles.
(PDF)

S4 Fig Representative gating for proliferation and activation markers. After gating on CD45^+^ singlets and excluding CD3^+^, CD14^+^ and CD20^+^ cells, as well as dead cells, NK cells were defined as CD8^+^CD3^-^ lymphocytes and verified by NKG2A staining. KIR3DL01^+^ versus KIR3DL05^+^ subsets were differentiated by staining with the anti-human KIR2D-specific antibody NKVFS1 and with Mamu-A1*002 _Gag71-79_ GY9 tetramer. The gates for the Ki-67^+^, CD69^+^ and HLA-DR^+^ positive NK cells were determined based on non-specific staining with isotype control antibodies for each marker.
(PDF)

S5 Fig CCR7 and CXCR3 expression on NK cells in response to SIV infection. Longitudinal changes in the expression of CCR7 (A) and CXCR3 (B) were monitored for KIR3DL01^+^, KIR3DL05^+^ and KIR3DL01^-^05^-^ NK cells. Absolute counts were calculated as a percentage of total NK cell counts by staining PBMCs with antibodies to CD3, CD8, NKG2A, KIR3DL01 and KIR3DL05 (tetramer), and to markers of lymph node homing (CCR7) and inflammation (CXCR3). The mean and standard error (error bars) are shown for each NK cell subset. Significance values for acute (weeks 1–4) and chronic (weeks 6–24) infection compared to pre-infection (week 0) are indicated with asterisks color-coded to the corresponding cell population (p<0.05*, mixed effects models).
(PDF)

S6 Fig Representative gating for CD107a and TNFα staining. After gating on CD3^-^ singlets and excluding dead cells, NK cells were defined as CD8^+^NKG2A^+^ lymphocytes. KIR3DL01^+^ versus KIR3DL05^+^ subsets were differentiated by staining with the anti-human KIR2D-specific antibody NKVFS1 and with Mamu-A1*002 _Gag71-79_ GY9 tetramer. CD107a^+^ and TNFα^+^ cells were gated as indicated, with CD107a staining used as counterstain for TNFα.
(PDF)

S7 Fig Representative gating for differentiating NK cell subsets in gut biopsies. Lymphocytes isolated from pooled biopsies of the colon and rectum were stained with antibodies to CD45, NKG2A, CD16, CD56 and KIR3DL01, and tetramer to KIR3DL05. NK cells were identified as NKG2A^+^CD45^+^ lymphocytes, and after excluding dead cells, KIR3DL01^+^ versus KIR3DL05^+^ and CD16^+^ versus CD56^+^ subsets were gated as indicated.
(PDF)

S1 Table GenBank accession numbers for newly identified KIR alleles. KIR genotyping was performed by next generation sequencing of cDNA reverse-transcribed from full-length mRNA transcripts. Immuno-Polymorphism Database designations and GenBank accession numbers for newly identified rhesus macaque *KIR* alleles are listed in the table.
(DOCX)
