# Supplementary figures and images for: KIR3DL01 upregulation on gut natural killer cells in response to SIV infection of KIR- and MHC class I-defined rhesus macaques
Source: PLoS Pathog. 2017 Jul 14;13(7):e1006506. doi: 10.1371/journal.ppat.1006506 (PMC5529027; doi:10.1371/journal.ppat.1006506)

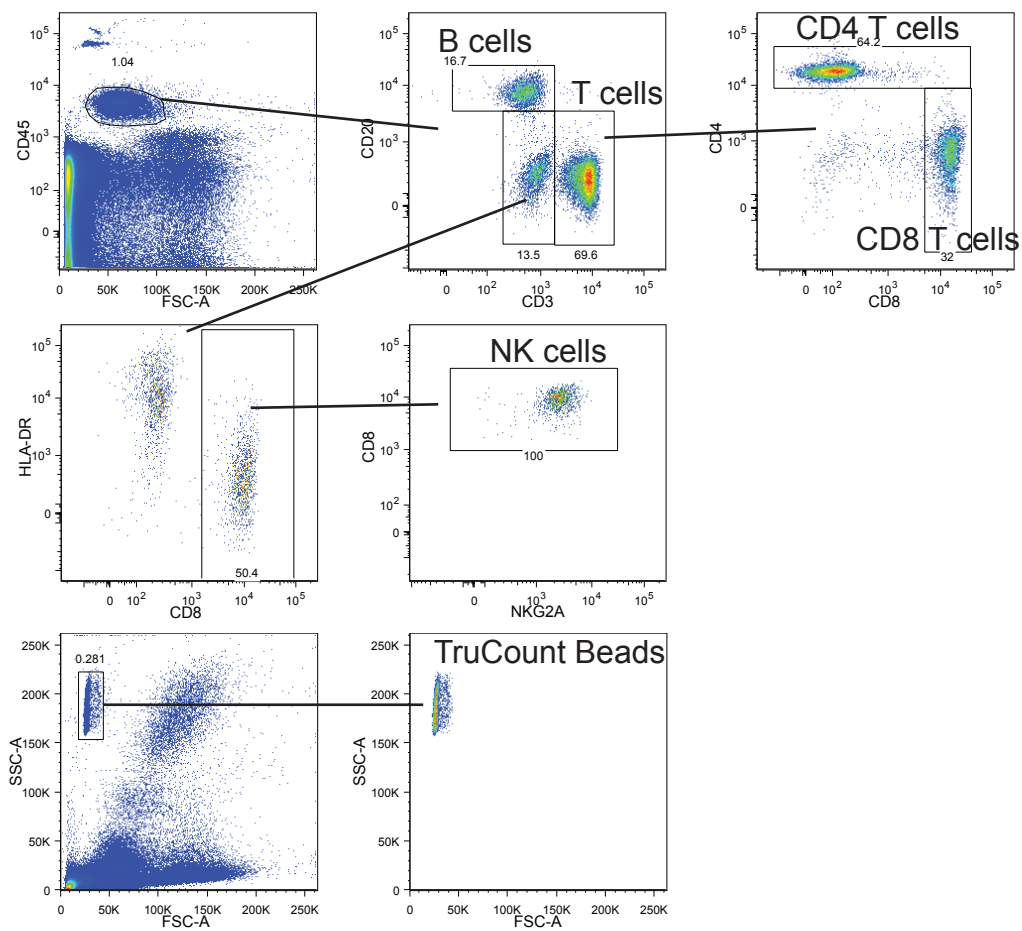

Supplement: S1 Fig — Whole blood was stained with antibodies to CD45, CD3, CD4, CD8, CD20, NKG2A (CD159a) and HLA-DR. CD45+ cells were subdivided into CD20+, CD3+ or CD20-CD3- lymphocytes. The CD3+ lymphocytes were further subdivided in CD4+ and CD8+ T cell subsets. NK cells were defined as CD45+CD3-CD8+ lymphocytes and further confirmed as NKG2A+. Gating for enumeration of the Trucount beads is shown in the bottom panels. (PDF) [file ppat.1006506.s001.pdf]

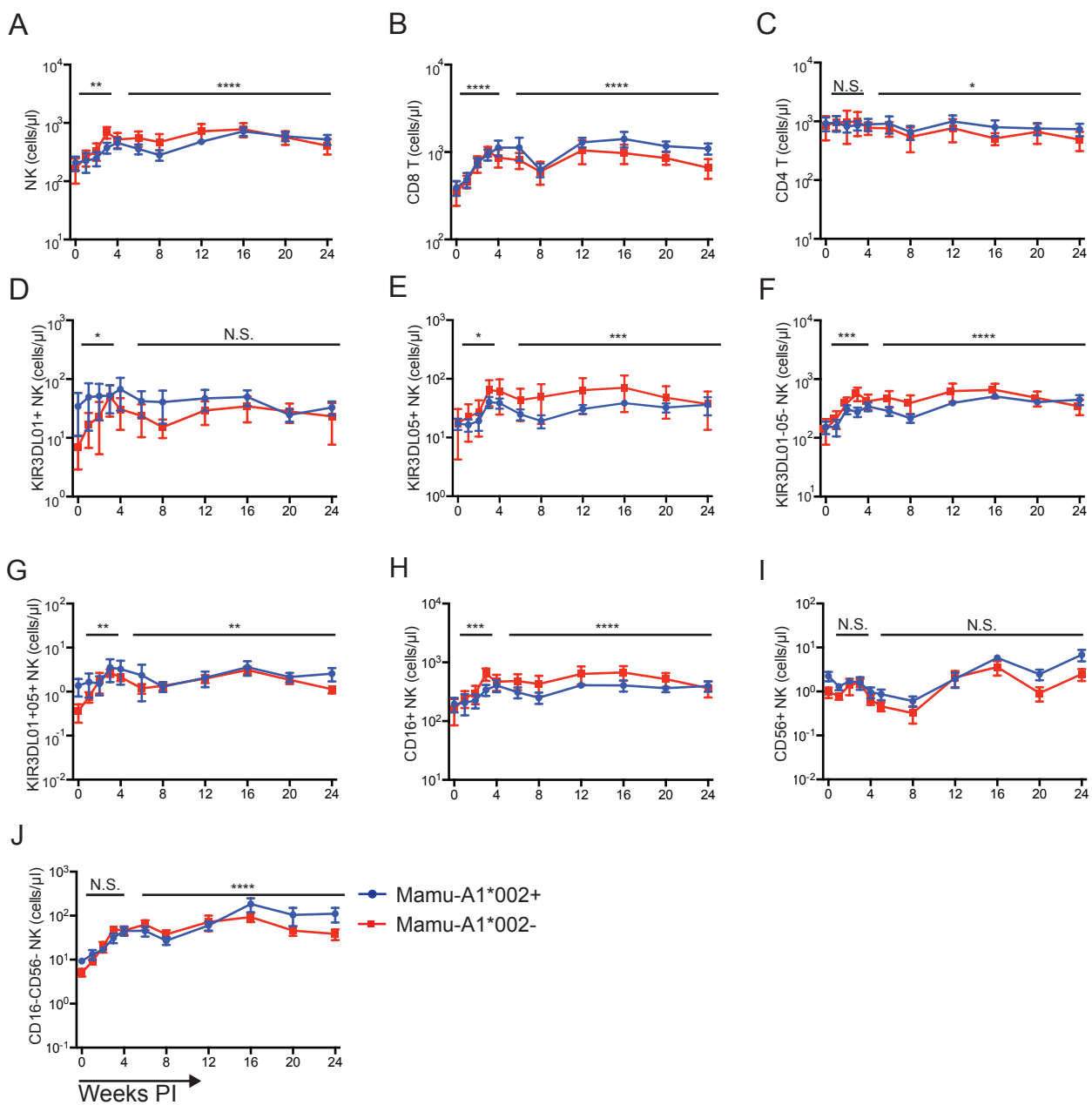

Supplement: S2 Fig — The means and standard error (error bars) of absolute counts for total NK cells (A), CD8+ T cells (B), CD4+ T cells (C), KIR3DL01+ NK cells (D), KIR3DL05+ NK cells (E), KIR3DL01-05- NK cells (F), KIR3DL01+05+ NK cells (G), CD16+ NK cells (H), CD56+ NK cells (I) and CD16-CD56- NK cells (J) are shown for Mamu-A1*002+ versus–A1*002- animals. Gating strategies for determining absolute lymphocyte counts in blood and the percentages of PBMCs expressing CD16, CD56, KIR3DL01 and KIR3DL05 are shown in S1 and S4 Figs. Statistics were calculated using a mixed effects model by comparing results from acute (week 1–4) and chronic (weeks 6–24) infection to pre-infection (week 0) (p<0.05 *, p<0.01**, p< 0.005*** & p<0.001****). (PDF) [file ppat.1006506.s002.pdf]

A

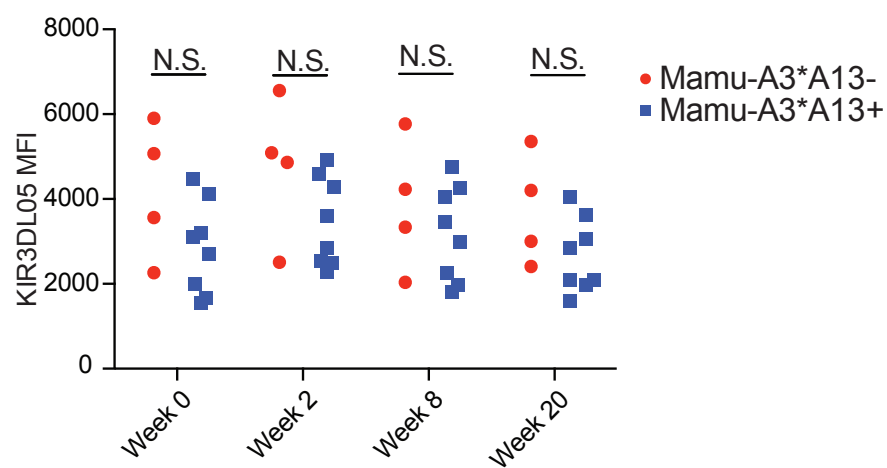

B

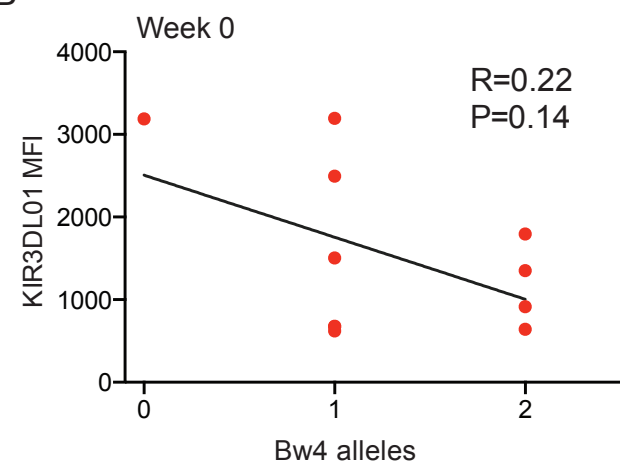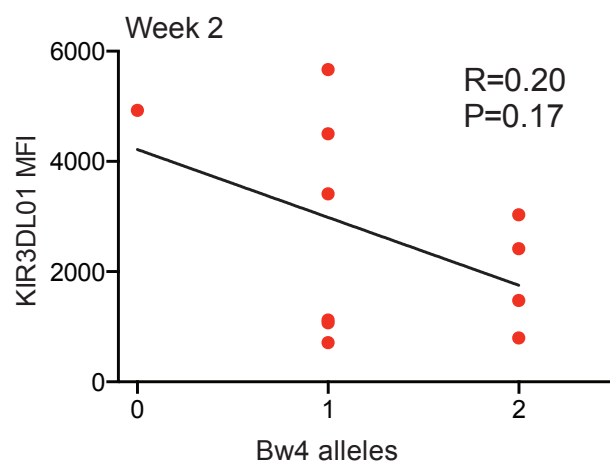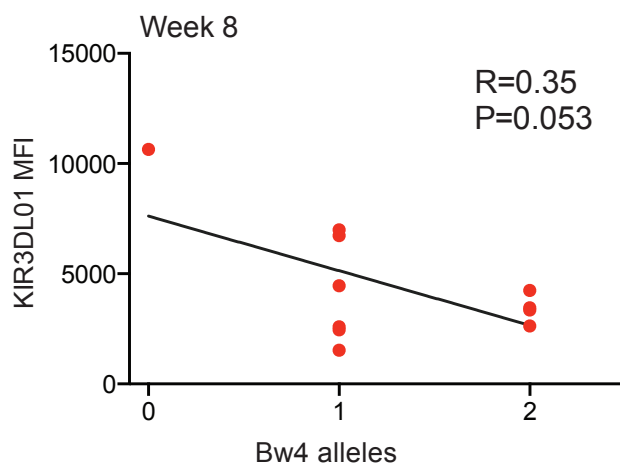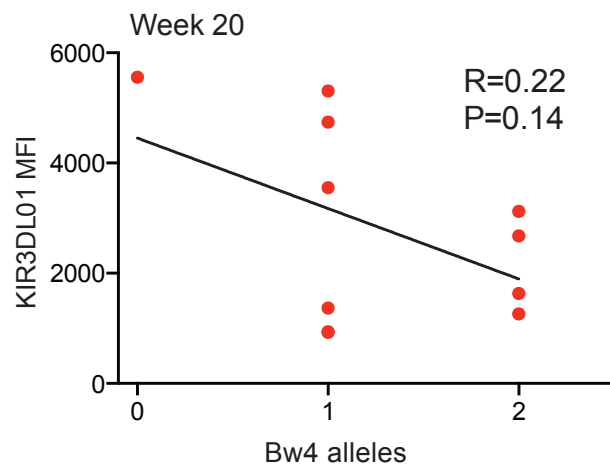

Supplement: S3 Fig — Comparison of the mean fluorescence intensity of KIR3DL05 staining on NK cells from Mamu-A13*13+ (blue) versus Mamu-A3*13- (red) animals prior to SIV infection (week 0) and at weeks 2, 8 and 20 post-infection (A). Differences in KIR3DL05 staining were not significant (N.S.) by Mann-Whitney U-test comparisons. Comparison of the mean fluorescence intensity of KIR3DL01 staining on NK cells versus the number of alleles predicted to encode Mamu-Bw4 ligands for this receptor prior to SIV infection (week 0) and at weeks 2, 8 and 20 post-infection (B). Linear regression analysis did not reveal significant correlations between KIR3DL01 staining and the number of Mamu-Bw4 alleles. (PDF) [file ppat.1006506.s003.pdf]

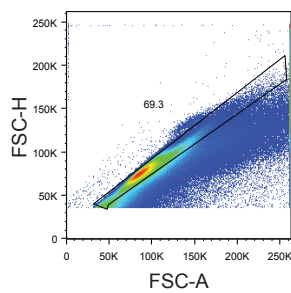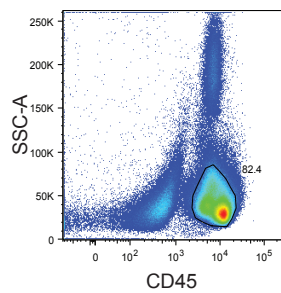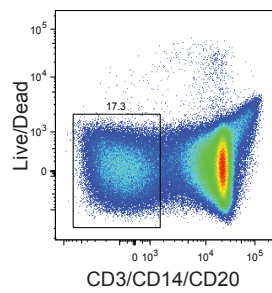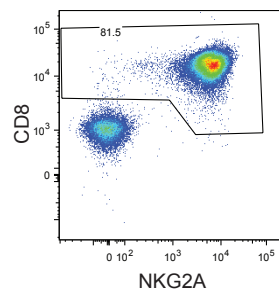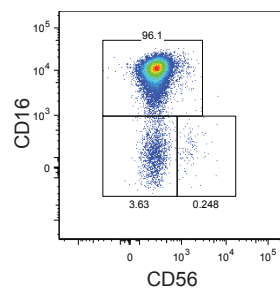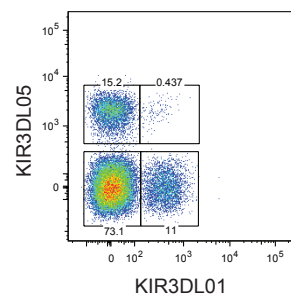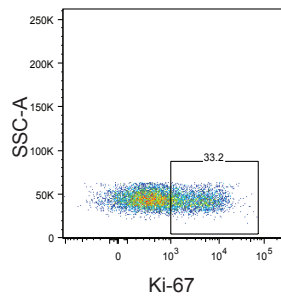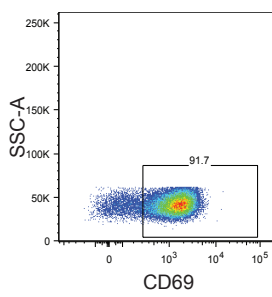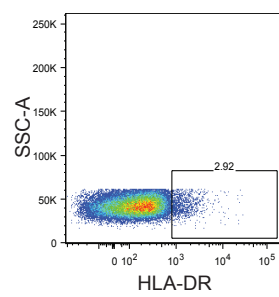

Supplement: S4 Fig — After gating on CD45+ singlets and excluding CD3+, CD14+ and CD20+ cells, as well as dead cells, NK cells were defined as CD8+CD3- lymphocytes and verified by NKG2A staining. KIR3DL01+ versus KIR3DL05+ subsets were differentiated by staining with the anti-human KIR2D-specific antibody NKVFS1 and with Mamu-A1*002 Gag71-79 GY9 tetramer. The gates for the Ki-67+, CD69+ and HLA-DR+ positive NK cells were determined based on non-specific staining with isotype control antibodies for each marker. (PDF) [file ppat.1006506.s004.pdf]

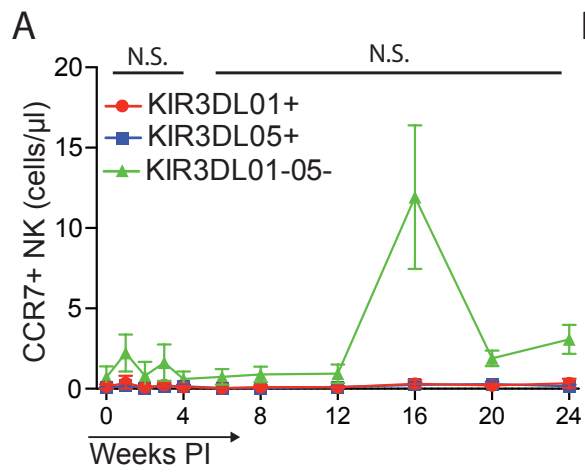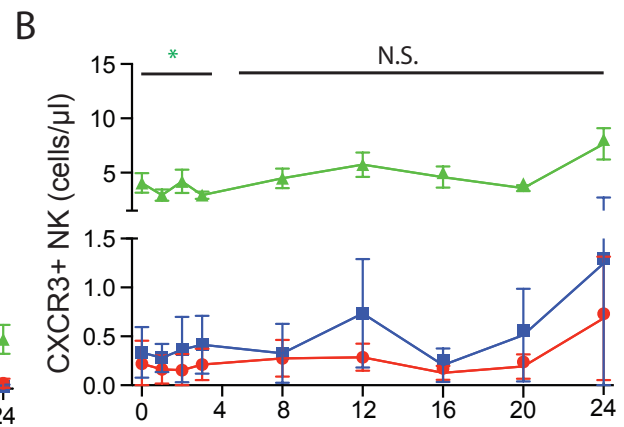

Supplement: S5 Fig — Longitudinal changes in the expression of CCR7 (A) and CXCR3 (B) were monitored for KIR3DL01+, KIR3DL05+ and KIR3DL01-05- NK cells. Absolute counts were calculated as a percentage of total NK cell counts by staining PBMCs with antibodies to CD3, CD8, NKG2A, KIR3DL01 and KIR3DL05 (tetramer), and to markers of lymph node homing (CCR7) and inflammation (CXCR3). The mean and standard error (error bars) are shown for each NK cell subset. Significance values for acute (weeks 1–4) and chronic (weeks 6–24) infection compared to pre-infection (week 0) are indicated with asterisks color-coded to the corresponding cell population (p<0.05*, mixed effects models). (PDF) [file ppat.1006506.s005.pdf]

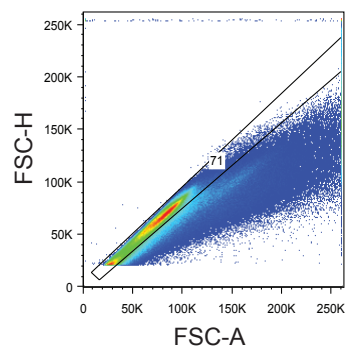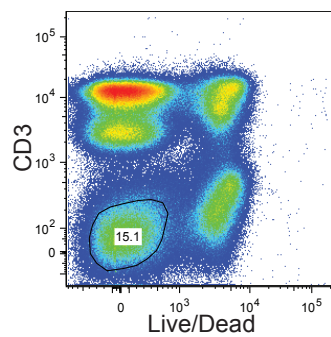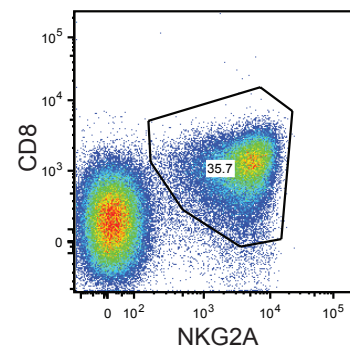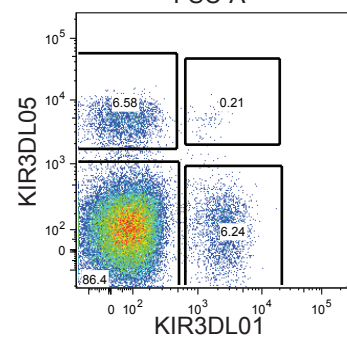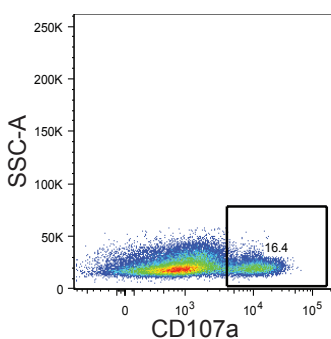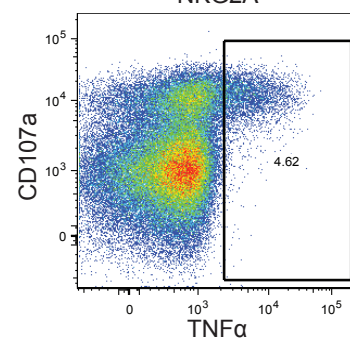

Supplement: S6 Fig — After gating on CD3- singlets and excluding dead cells, NK cells were defined as CD8+NKG2A+ lymphocytes. KIR3DL01+ versus KIR3DL05+ subsets were differentiated by staining with the anti-human KIR2D-specific antibody NKVFS1 and with Mamu-A1*002 Gag71-79 GY9 tetramer. CD107a+ and TNFα+ cells were gated as indicated, with CD107a staining used as counterstain for TNFα. (PDF) [file ppat.1006506.s006.pdf]

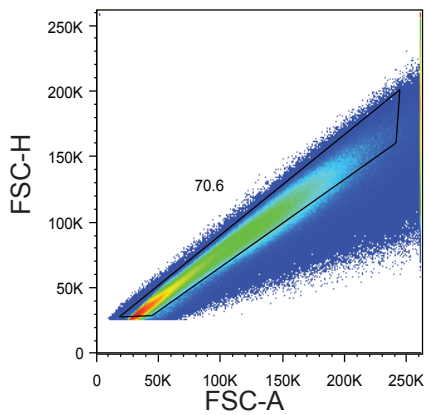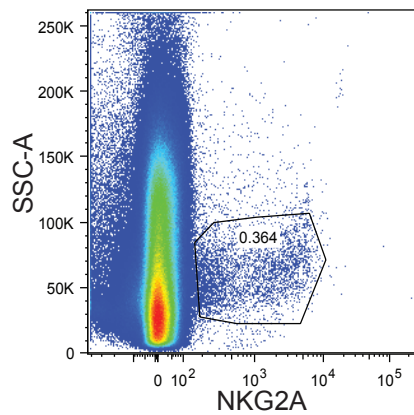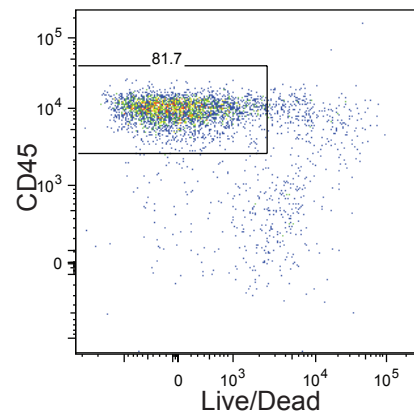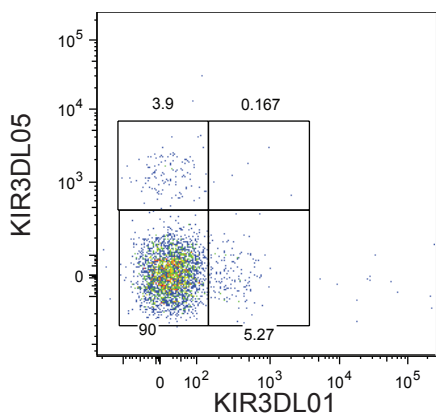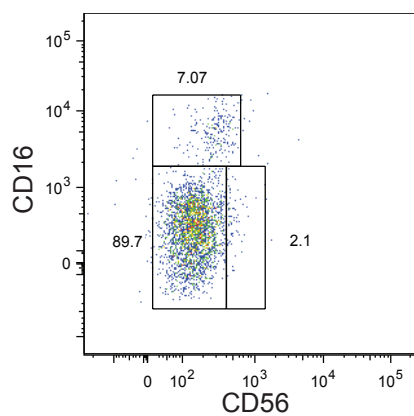

Supplement: S7 Fig — Lymphocytes isolated from pooled biopsies of the colon and rectum were stained with antibodies to CD45, NKG2A, CD16, CD56 and KIR3DL01, and tetramer to KIR3DL05. NK cells were identified as NKG2A+CD45+ lymphocytes, and after excluding dead cells, KIR3DL01+ versus KIR3DL05+ and CD16+ versus CD56+ subsets were gated as indicated. (PDF) [file ppat.1006506.s007.pdf]
