# Supplementary material for: KIR3DL01 upregulation on gut natural killer cells in response to SIV infection of KIR- and MHC class I-defined rhesus macaques
Source: PLoS Pathog. 2017 Jul 14;13(7):e1006506. doi: 10.1371/journal.ppat.1006506 (PMC5529027; doi:10.1371/journal.ppat.1006506)
Supplement: S1 Table — KIR genotyping was performed by next generation sequencing of cDNA reverse-transcribed from full-length mRNA transcripts. Immuno-Polymorphism Database designations and GenBank accession numbers for newly identified rhesus macaque KIR alleles are listed in the table. (DOCX) [file ppat.1006506.s008.docx]

**Table S1. GenBank accession numbers of newly identified KIR alleles**

| **Designation** | **Accession Number** |
| --- | --- |
| Mamu-KIR1D*002 | MF164921 |
| Mamu-KIR1D*003:01 | KY660349 |
| Mamu-KIR1D*003:02 | FJ217804 |
| Mamu-KIR2DL04*021 | KY660351 |
| Mamu-KIR2DL04*022 | KY660352 |
| Mamu-KIR2DL04*023 | KY660353 |
| Mamu-KIR2DL04*012 | KY660350 |
| Mamu-KIR2DL05*025 | KY660354 |
| Mamu-KIR2DL05*026 | KY660355 |
| Mamu-KIR2DL05*027 | KY794599 |
| Mamu-KIR3DL01*002 | MF164922 |
| Mamu-KIR3DL05*015 | KY660356 |
| Mamu-KIR3DL05*016 | KY660357 |
| Mamu-KIR3DL07*024 | MF164923 |
| Mamu-KIR3DS01*005 | KY660358 |
| Mamu-KIR3DS02*015:01 | KY660366 |
| Mamu-KIR3DS02*015:02 | KY660367 |
| Mamu-KIR3DS03*004 | KY660359 |
| Mamu-KIR3DS03*001:01 | NM0013118349 |
| Mamu-KIR3DS04*007 | KY660360 |
| Mamu-KIR3DS04*008 | KY660361 |
| Mamu-KIR3DS04*001:02 | JN613294 |
| Mamu-KIR3DS05*006 | KY660362 |
| Mamu-KIR3DS05*004 | KY660365 |
| Mamu-KIR3DS06*010 | KY660363 |
| Mamu-KIR3DS06*011 | MF164925 |
| Mamu-KIR3DSw07*003 | KY660364 |

KIR genotyping was performed by next generation sequencing of cDNA reverse-transcribed from full-length mRNA transcripts. Immuno-Polymorphism Database designations and GenBank accession numbers for newly identified rhesus macaque *KIR* alleles are listed in the table.
